# Establishing an invertebrate *Galleria mellonella* greater wax moth larval model of *Neisseria gonorrhoeae* infection

**DOI:** 10.1080/21505594.2021.1950269

**Published:** 2021-07-25

**Authors:** Aiste Dijokaite, Maria Victoria Humbert, Emma Borkowski, Roberto M La Ragione, Myron Christodoulides

**Affiliations:** a Neisseria Research Group, Molecular Microbiology, Academic School of Clinical and Experimental Sciences, University of Southampton Faculty of Medicine, Southampton, UK; b Department of Pathology and Infectious Diseases, School of Veterinary Medicine, Faculty of Health and Medical Sciences, University of Surrey, Guildford, UK

**Keywords:** *Neisseria gonorrhoeae*, *Galleria mellonella*, infection, histopathology

## Abstract

*Neisseria gonorrhoeae* (gonococcus) causes the human sexually transmitted disease gonorrhea. Studying gonococcal pathogenesis and developing new vaccines and therapies to combat the increasing prevalence of multi-antibiotic resistant bacteria has made use of many *ex vivo* models based on human cells and tissues, and *in vivo* vertebrate models, for example, rodent, pig and human. The focus of the current study was to examine the utility of the invertebrate greater wax moth *Galleria mellonella* as an *in vivo* model of gonococcal infection. We observed that a threshold of ~10^6^ – 10^7^ gonococci/larva was required to kill >50% of larvae (P < 0.05), and increased toxicity correlated with reduced health index scores and pronounced histopathological changes such as increases in the total lesion grade, melanized nodules, hemocyte reaction, and multifocal adipose body degeneration. Larval death was independent of the expression of pilus or Opa protein or LOS sialylation within a single gonococcal species studied, but the model could demonstrate relative toxicity of different isolates. *N. meningitidis, N. lacatamica* and gonococci all killed larvae equally, but were significantly less toxic (P > 0.05) than *Pseudomonas aeruginosa*. Larvae primed with nontoxic doses of gonococci were more susceptible to subsequent challenge with homologous and heterologous bacteria, and larval survival was significantly reduced (P < 0.05) in infected larvae after depletion of their hemocytes with clodronate-liposomes. The model was used to test the anti-gonococcal properties of antibiotics and novel antimicrobials. Ceftriaxone (P < 0.05) protected larvae from infection with different gonococcal isolates, but not azithromycin or monocaprin or ligand-coated silver nanoclusters (P > 0.05).

## Introduction

*Neisseria gonorrhoeae* (gonococcus) causes the human sexually transmitted disease gonorrhea. Worldwide, there are ~87 million cases of gonococcal infection reported annually by the World Health Organization (WHO) [1], with the majority in the least developed and low-to-middle-income countries. The gonococcus infects the mucosal epithelium of the male urethra, causing urethritis and painful discharge, and the female endo/ectocervix, causing a mucopurulent cervicitis. However, in approximately 10–25% of untreated women, gonococci can ascend into the upper reproductive tract and cause pelvic inflammatory disease, which can result in long-term and/or permanent chronic pelvic pain, fallopian tube damage, endometritis, ectopic pregnancy, and infertility [2]. Furthermore, rare Disseminated Gonococcal Infection (DGI) can be manifested as septicemia, meningitis, arthritis, tenosynovitis, endocarditis and cutaneous infection. Other body sites can be infected, with reports of gonococcal oro- and nasopharyngeal infections, tonsillitis, stomatitis, parotitis, scalp abscess, and mastitis [3]. Gonococcal conjunctivitis is still reported in the least developed and low-to-middle-income countries and adult gonococcal keratoconjunctivitis is also now seen as a serious emerging infection. Furthermore, co-infection with other sexually transmitted disease pathogens, for example, HIV, *Treponema pallidum, Trichomonas,* and *Chlamydia*, is common, and gonococci are known to increase the risk of HIV transmission and infection.

There is a substantial literature on *N. gonorrhoeae* pathogenesis studies using many *ex vivo* models based on human cells and tissues, mouse models, a pig model, and the experimental human male volunteer urethral gonorrhea model [4–9]. These models have informed considerably our understanding of gonococcal–host interactions, which is central to developing vaccines and new therapies to combat the increasing prevalence of multi-antibiotic resistant gonococci [10]. Testing in vertebrate mouse models is a pre-requisite to human clinical trials, but they are expensive for high-throughput studies and require considerable operator experience. Complementary *in vivo* models could make use of invertebrates and the utility of the greater wax moth *Galleria mellonella* (Subfamily *Galleriinae*, Family *Pyralidae*, Order *Lepidoptera*) [11] is the focus of our study. *G. mellonella* is a pest to honey bee colonies and undergoes complete metamorphosis, moving from egg to larva to pupa to adult [12]. *G. mellonella* larvae possess several properties that make them useful for studying microbial infection [13]: they are readily available and inexpensive to purchase, do not require special equipment or conditions of storage for short period of times, their size makes injection, homogenization and hemolymph extraction facile, they have a short life cycle making them suitable for large-scale studies, they can survive at between 15°C and 37°C, their genome has been sequenced and their use does not require ethical approval. In addition, their innate immune system shares many similarities with the mammalian innate immune system. Cellular immunity is mediated by hemocytes that function in phagocytosis, nodulation, and encapsulation [14]. Other immunity factors include melanization [15], the prophenolaxidase activating system (Pro-PO-AS) [16], the synthesis of small antimicrobial peptides [17] and lysozyme [18].

There is extensive literature describing the use of *G. mellonella* to study infection by many bacteria [13], for example *Pseudomonas aeruginosa* [19], *Mycobacterium tuberculosis* [20], *Burkholderia spp* [21], *Staphylococcus aureus* [22], *Listeria monocytogenes* [23], *Streptococcus pneumoniae* [24], *Actinobacillus pleuropneumoniae* [25], *Shigella spp* [26], *Enterobacter cloacae* [27], *Bacillus thuringiensis* [28], and fungi, such as *Candida* [29] and parasites, such as *Leishmania* and *Trypanosoma spp* [30]. In addition, *G. mellonella* is being used as a model for testing new antibacterial, antifungal, and anti-parasite strategies and novel drugs and antibiotics [31]. In the current study, we describe the development of a *G. mellonella* larval model of *N. gonorrhoeae* infection, which to our knowledge has not been previously reported for this bacterium.

## Materials and methods

### Bacteria and growth media

Information on the *Neisseria gonorrhoeae* strains and other bacteria used in this study is provided in Table 1. *Neisseria spp*. and *P. aeruginosa* were grown on supplemented GC agar plates [32] or in supplemented GC broth incubated at 37°C in an atmosphere containing 5% (v/v) CO_2_. *Lactobacillus spp*. were cultured on Tryptic Soy Agar (Oxoid) at 37°C with 5% (v/v) CO_2_.

**Table 1.** *Neisseria gonorrhoeae* strains and other bacteria used in this study

Organism	Isolate	Description	Reference
*Neisseria gonorrhoeae*	P9-17	A Pil^+^ Opa_b_^+^ variant of *N. gonorrhoeae* strain P9, a 1B-26 serovar isolate originally isolated from a patient with gonococcal prostatitis in London, UK.	[80]
	P9-1	Pil^−^ Opa^−^ variant	[81]
	P9-2	Pil^+^Opa^−^ variant	[81]
	P9-16	Pil^−^Opa^+^ variant	[81]
	AR Isolate Bank	Centers for Disease Control and Prevention (CDCP)/Food and Drug Administration (FDA) Antibiotic/Antimicrobial Resistance Isolate Bank of 50 *N. gonorrhoeae* isolates https://www.cdc.gov/drugresistance/resistance-bank/currently-available.html.	
*Neisseriameningitidis*	MC58	B:15:P1.7,16b isolate from an outbreak of meningococcal infection that occurred in Stroud, Gloucestershire in the mid-1980’s.	[82]
*Neisseria lactamica*	Y92–1009	Originally isolated during a school carriage study in Northern Ireland in 1992.	[83]
*Pseudomonas aeruginosa*	PAO1	Holloway1C Stanier131, obtained from the National Collection of Industrial, Food and Marine Bacteria, UK.	
*Lactobacillus crispatus*	-	ATCC338820	
*Lactobacillus gasseri*	-	NCTC13722	
*Lactobacillus brevis*	-	NCTC13386	

Killed gonococci were prepared by heating at 56°C for 60 min in a water bath. Sialylation of gonococcal lipooligosaccharide (LOS) glycans was done by growing bacteria on Cytidine-5′-monophospho-N-acetylneuraminic acid (CMP-NANA, Merck) supplemented plates. Briefly, 2 mg of CMP-NANA powder was dissolved in 1 mL of ultra-high quality (UHQ) water and the solution spread onto the surface of 20 mL of supplemented GC agar medium to give a final concentration of 50 µg/mL. Gonococci were grown directly onto CMP-NANA plates for use. Vancomycin, Colistin sulfate, Nystatin, and Trimethoprim (VCNT, Oxoid) supplemented GC agar plates were used to selectively isolate gonococci from infection experiments with mixed bacteria and to recover viable gonococci from the hemolymph extraction. Powdered VCNT was dissolved aseptically with 2 mL of sterile UHQ and mixed with 500 mL of GC agar medium to give final concentrations per 20 ml agar plate of V 0.06 mg, C 0.15 mg, N 250 IU and T 0.1 mg.

### *Preparation of* N. gonorrhoeae *lipooligosaccharide (LOS)-replete outer membranes (OM)*

OM of *N. gonorrhoeae* strain P9-17 were prepared by lithium acetate extraction as described previously [33]. Protein concentration was determined using a Bicinchoninic acid (BCA) assay, following the manufacturer’s instructions (Thermo Fischer).

### *Preparation of live microbial inocula for infection of* G. mellonella

*N. gonorrhoeae, P. aeruginosa,* and *Lactobacillus spp*. were cultured from frozen stocks onto appropriate agar media for ≤16 h at 37°C with 5% (v/v) CO_2_. On the next day, single colonies were selected from the overnight culture and spread onto fresh agar plates, which were incubated at 37°C with 5% (v/v) CO_2_ for 6 h. Then, the culture from the agar plates was suspended in supplemented GC broth. Absorbance (Optical Density, OD) of the bacterial suspensions was measured by spectrophotometer at λ595 nm (iMark microplate reader, BIO-RAD). When the required optical density was reached for the microbial inocula, 10 µL of each different dose were pipetted onto a sterile Petri dish 11 times to generate 11 discrete bubbles. In addition, 10 µL of titrated microbial inocula were pipetted onto fresh agar plates in triplicate and grown overnight to determine concentrations of inocula (colony forming units (CFU)/mL).

### *Injection of* G. mellonella *larvae*

Last larval stage *Galleria* larvae (Wazp-Brand, Monkfield Nutrition, UK) was collected from a local pet shop immediately following delivery to assure fresh insects. Larva were examined visually and sorted by size and each larva was weighed and larva of 0.25–0.35 g were used for the model. Larvae (n = 10) were placed into individual sterile Petri dishes and each test or control group had at least three sets of Petri dishes with larvae (n = 30). Larvae were handled gently and with care and maintained at 37°C with a 5% (v/v) CO_2_ atmosphere for the duration of the experiments.

Initial experiments were done to determine which medium was nontoxic toward *Galleria mellonella* larvae and maintained bacterial viability. Larvae were injected (10 µL/larva) with phosphate-buffered saline (PBS), pH 7.4, PBS containing the GC supplements [32], PBS with 10 mM of ferric citrate, PBS with 10 mM of ferric nitrate, or with supplemented GC broth alone.

Groups of 10 individual larvae were injected in the last left pro-leg with 10 µL of various doses of live bacteria, dead bacteria, or OM using U-100 insulin syringes (BB Micro-Fine). A single syringe was used for the 10 caterpillars within the same group. Control groups for each experiment included 1) untreated larvae; 2) trauma – larvae were pricked once in the last left pro-leg with a sterile syringe; 3) sterile supplemented GC broth; and 4) PBS. In all experiments in which gonococci and other bacteria were tested, a positive control was included of larvae that were injected with 10 µL of supplemented GC broth containing ~150 CFU/larva of *P. aeruginosa* PAO1. This control group always resulted in all larvae dying within the first 16 hours of the experiment.

At the end of each experiment, both dead and surviving *G. mellonella*, were placed into plastic bags and kept at −20°C overnight to sedate and kill them humanely. On the following day, the plastic bag containing dead larvae was autoclaved at 121°C for 15 min, 2.68 kg/cm^2^ and discarded.

### G. mellonella *Health Index Scoring System (HISS)*

A *G. mellonella* Health Index Scoring System (HISS) based on Loh *et al*. [34] was used to observe and record larval health changes (Table 2). The virulence of strain P9-17 was measured at 0 h, 16 h, 24 h, 40 h, and 48 h. The development of melanization was observed, and larvae were recorded as dead when they stopped responding to stimuli, for example touching of larvae with disinfected tweezers or movement in response to tapping of the Petri dish.

**Table 2.** ***Galleria mellonella* Health Index Scoring System (HISS)**. A healthy, uninfected waxworm typically scores between 9 and 10, and an infected, dead wax worm typically scores 0. The health index scores from a very early stage of post-infection correlate well with the infectious dose, with higher inocula generally resulting in lower health indices. Based on Loh *et al*. [34]

Category	Description	Score
Activity	no movement	0
	minimal movement on stimulation	1
	move when stimulated	2
	move without stimulation	3
Cocoon formation	no cocoon	0
	partial cocoon	0.5
	full cocoon	1
Melanization	black larvae	0
	black spots on brown larvae	1
	≥3 spots on beige larvae	2
	<3 spots on beige larvae	3
	no melanization	4
Survival	dead	0
	alive	2

### *Co-infection of* G. mellonella *with* Lactobacillus spp. *and* N. gonorrhoeae

The method was adapted from Santos *et al*. [35] and Scalfaro *et al*. [36]. *Lactobacillus brevis, crispatus*, and *gasseri* were cultured as described above for live microbial inocula. Initially, *G. mellonella* sensitivity assay was done by injecting different dilutions (OD 0.1 (~10^5^), 0.6 (~10^6^), 0.8 (~10^7^) and 1.0 (~10^8^) CFU/larva) of all three *Lactobacillus* species into larvae and monitoring their survival for 48 h.

After assessing sensitivity, *L. gasseri* and *N. gonorrhoeae* P9-17 were cultured as described above and 10 larvae per group were each injected with 10 µL of supplemented GC broth containing either 0.1 (~10^5^ CFU/larva), 0.6 (~10^6^ CFU/larva) or 0.8 OD (~10^7^ CFU/larva) of *L. gasseri* bacterial suspension or 0.8 OD (~10^7^ CFU/larva) of P9-17. After 16 h, groups of *L. gasseri*-primed larvae were injected with 10 µL supplemented GC broth of 0.8 OD of P9-17 bacterial suspension. Larvae were monitored for 48 h and dead larvae were counted at 24 and 48 h post-infection with P9-17.

### *Effect of priming* G. mellonella *with* N. gonorrhoeae

The method was adapted from Bergin *et al*. [37] and Taszlow *et al*. [38]. *N. gonorrhoeae* P9-17 was cultured on supplemented GC agar and in broth as described above for live microbial inocula. Fifteen larvae were infected with 10 µL of 0.1 OD (~0.7 × 10^5^ CFU/larva), which is not lethal to *G. mellonella*. After 16 h, a high dose of 10 µL of 0.8 OD (10^7^ CFU/larva), medium dose of 10 µL of 0.4 OD (10^6^ CFU/larva) and low dose of 10 µL of 0.1 OD (10^5^ CFU/larva) was used to infect larvae. We also tested the impact of priming the larvae with 0.1 OD of P9-17 followed by infection with 0.8 OD of live *L. gasseri* and 0.8 OD of heat-inactivated *L. gasseri* and P9-17. Survival of larvae was assessed for 48 h post-re-infection with bacteria.

### *Recovery of viable* N. gonorrhoeae *from* G. mellonella

Initially, we trialed several methods to quantify gonococcal numbers within infected larvae, including hemolymph recovery using pestle and mortar, flow cytometry with P9-17 expressing Green Fluorescent Protein, and a LIVE/DEAD BacLight Bacterial Viability Kit, but none worked as reliably (data not shown) as the recovery of gonococci from the hemolymph of larvae infected with gonococci using VCNT selective agar plates. Briefly, larvae were placed at −20°C for 2 to 4 min and then transferred to a Class II biosafety cabinet. A sterile scalpel blade was used to cut 2–3 mm from the larval end and hemolymph was squeezed into 1.5 mL Eppendorf tubes. Extracted hemolymph was diluted serially in sterile supplemented GC broth and 10 µL aliquots spread onto VCNT supplemented GC agar plates in triplicate and incubated overnight at 37°C with 5% (v/v) CO_2_. CFU were counted and recorded after 16–48 h of incubation. Hemolymph extractions were done at 16 h, 24 h, 40 h, and 48 h post-injection of bacteria into *G. mellonella*.

### *Chemical depletion of* G. mellonella *hemocytes*

Dichloromethylene-bisphosphonate (clodronate) is a chemical used to deplete macrophages [39] and was used in our study to deplete hemocytes from *G. mellonella*. A suspension of artificially prepared lipid vesicles encapsulating clodronate was used for the experiments and adapted from the method of Kwon and Smith, who used clodronate-filled liposomes to deplete hemocytes in *Anopheles gambiae* mosquitos [40]. Liposomes were from Liposoma research (https://clodronateliposomes.com), and two types of liposomes were used: control liposomes (Lip) in PBS and liposomes containing 5 mg/mL clodronate (CDLip). Groups of 10 *G. mellonella* larvae were injected in the last left pro-leg with Lip (10 µL) or CDLip (10 µL) at neat and at 1/5, 1/10 and 1/20 dilutions. After 16 h, the larvae were injected with various doses of *N. gonorrhoeae* P9-17 (OD 0.1, or 0.4 or 0.8) and larval survival was monitored over a 48 h period. Neither Lip nor CDLip killed gonococci when tested against the organism alone.

### Histopathology

Live larvae were fixed at 2, 16, and 24 h to examine cellular immune responses to gonococcal infection. Larvae infected with *N. gonorrhoeae* P9-17 and control, uninfected larvae were fixed by injecting 100 µL of 10% (v/v) of neutral buffered formalin (CellPath) into the last right pro-leg of the caterpillar and immersing three larvae per time point in a labeled Cellstor Pot filled with 60 mL of 10% (v/v) of neutral buffered formalin (CellPath). Injection and fixation were repeated three times to generate larvae for histopathology from three independent experiments.

Larvae were sectioned to produce longitudinal or transverse sections, placed into cassettes and then processed overnight using a Sakura VIP 6 Vacuum Infiltration Processor. Tissues were embedded into molten paraffin wax using Sakura TEC 5 Tissue Embedding Console. The blocks were cut using a Leica RM2235 microtome at 3 microns and sections placed onto regular glass slides. Once the sections were dry, they were placed into the Sakura DRS 2000 with Dryer where they were stained using a standard Hematoxylin & Eosin (H&E) protocol with xylene and alcohol washes. Following staining, the slides were cover-slipped using a xylene-based mountant and left to dry for 12 h and then examined using light microscopy.

Longitudinal sections were generated by bisecting the larva and embedding both halves in paraffin with the cut surface toward the microtome blade, yielding two sections of each larva for examination. For transverse-sectioned larvae, sections were generated as follows: the larva was first bisected transversely into equal halves and both the cranial and caudal halves were sectioned transversely into three equal segments. These were then embedded in paraffin to produce blocks with two cranial cut surfaces, two mid-body cut surfaces, and two caudal cut surfaces oriented toward the microtome blade, yielding a total of six transverse sections of each larva for histopathological examination. Quality of fixation in all slides was excellent and section quality ranged from fair to excellent. A histological grading scheme for common lesions was used to score larvae (Table 3).

**Table 3.** Histopathology grading scheme for common lesions in *Galleria mellonella.*

Lesion	Score	Description
**Melanization** **criteria**	0	No melanized nodules
	1	Melanized nodules occupying less than 10% of the coelomic cavity
	2	Melanized nodules occupying 10–20% of the coelomic cavity
	3	Melanized nodules occupying > 20% of the coelomic cavity
**Adipose body necrosis**	0	No necrosis
	1	Focal or multifocal necrosis affecting less than 10% of the tissue
	2	Focal or multifocal necrosis affecting 10–20% of the tissue
	3	Focal or multifocal necrosis affecting > 20% of the tissue
**Hemocyte** **reaction**	0	Low numbers of individual hemocytes within the coelom, monolayer lining subcuticular space (normal)
	1	Increased numbers of individual hemocytes, no distinctive clusters
	2	Formation of discrete hemocyte clusters in the subcuticular space and/or coelom
	3	Hemocyte clusters filling the subcuticular space and/or space between coelomic organs

### *Utility of* G. mellonella *for testing antimicrobial agents against* N. gonorrhoeae

The method was adapted from Ignasiak and Maxwell [41]. Standard microbial inocula (~8 × 10^7^ CFU/larva, that is, 10 µL of 0.8 OD) of *N. gonorrhoeae* P9-17 and isolates selected from the CDC/FDA panel were prepared as described above. The *G. mellonella* infection model was used to assess the ability of antibiotics used to treat gonorrhea, and novel antimicrobials, to prevent larval death over time. Ceftriaxone (sodium salt, Merck), was dissolved in PBS and then diluted for testing (10 µL/larva of 0.008 µg/mL to 1000 µg/mL). Azithromycin (Merck) was dissolved in dimethyl sulfoxide and then diluted with PBS for testing (10 µL/larva of 256 µg/mL to 1.024 mg/mL). 5-Mercapto-2-nitrobenzoic Acid-Coated Silver Nanoclusters [42] were diluted in PBS for testing (10 µL/larva of 0.467–58.44 µM). 1-Decanoyl-rac-glycerol (monocaprin, Merck) [43] was dissolved in 100% (v/v) ethanol and then diluted with PBS for testing (10 µL/larva of 0.1–50 mM).

The toxicity of antibiotics and antimicrobials toward *G. mellonella* was assessed by injecting larvae with 10 µL of different doses of the compounds and monitoring survival for 48 h. To assess their activity against gonococci *in vivo, G. mellonella* larvae (n = 10 per sample dose group) were injected with 10 µL of gonococcal standard dose (~8 × 10^7^ CFU/larva) and 10 µL of different doses of antimicrobial compound. Larval survival was measured at 16, 24, 40, and 48 h post-injection.

### *Minimum Inhibitory Concentration (MIC) values of ceftriazone and azithromycin for* N. gonorrhoeae *P9-17*

The broth microdilution method (based on the Clinical and Laboratory Standards Institute (CLSI) guidelines with some modifications as used by Foerster *et al.* [44]) was used to determine MIC values. Briefly, fresh 6 h cultures of P9-17 were grown as described above and 190 μL of a suspension of 5 × 10^5^ CFU/mL in supplemented GC broth was added to individual wells of a sterile 96 well microtiter plate. Next, 10 μL aliquots of the doses of antibiotic in 2-fold dilutions were added to triplicate wells. Controls were bacteria with no antibiotic and medium alone. Inocula were counted on supplemented GC agar plates as described above. The plates were incubated for 24 h at 37°C and optical density was read at λ595 nm (iMark microplate reader, BIO-RAD). The MIC value was defined as the minimum dose (μg/mL) that showed >90% reduction in optical density compared to the untreated control. Data are from triplicate-independent experiments with each antibiotic.

## Data analysis and reproducibility

Details of all technical and biological repeats for all of the experiments are reported in the Figure Legends. Data were analyzed using GraphPad Prism with unpaired t-Test to compare between groups, with P values <0.05 considered significant.

## Results

### *Effect of different growth media on* G. mellonella *survival*

Initially, we determined the optimal medium to use in our model that was not toxic to the larvae whilst maintaining gonococcal viability. Untreated larvae, traumatized larvae, and larvae injected with supplemented GC broth or PBS with and without the supplements all showed <10% mortality over a 72 h period (Figure S1). By contrast, larvae injected with PBS containing ferric nitrate or ferric citrate showed significant average mortality of 30–40% over 72 h, compared with untreated controls (P < 0.01). Although there was no significant difference between using supplemented GC broth or PBS for maximal larval survival (P > 0.05), we chose the former for all subsequent infection experiments since this medium is preferred for gonococcal growth and viability.

### *Infection of* G. mellonella *with* N. gonorrhoeae *strain P9-17*

**Figure 1.**
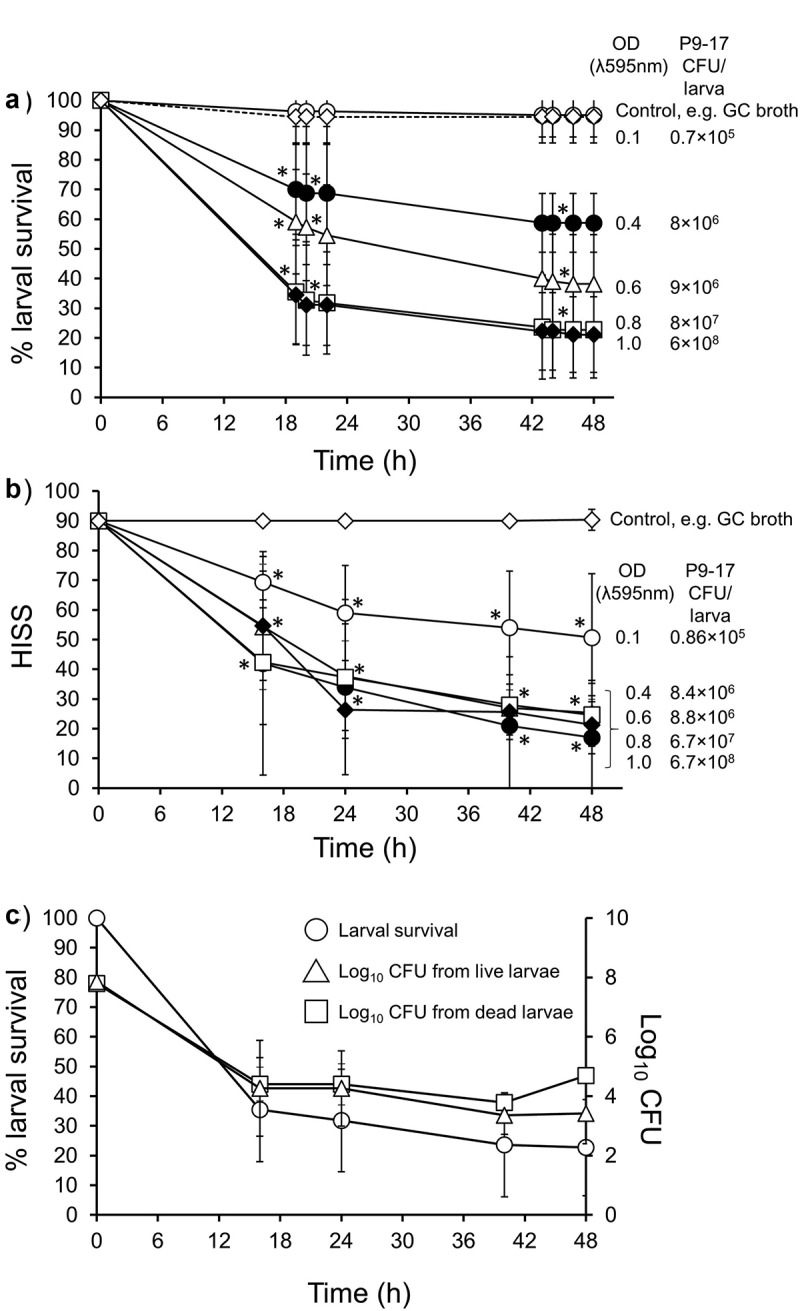
**Infection of *Galleria mellonella* with *Neisseria gonorrhoeae* strain P9-17. A)** The effect of varying inoculum dose of *N. gonorrhoeae* on survival of *G. mellonella*. Healthy larva (n = 10 per group) were infected with differing inocula of gonococci, incubated at 37°C and examined for survival by response to touch over a period of 48 h. The symbols represent mean survival from a minimum of 8 independent experiments and the error bars represent the standard deviation of the mean. Unpaired t test was done at each time point to compare survival in infected groups with control group of larvae injected with GC broth. * denotes statistically significant difference for all data points with P < 0.001. **B)** Health Index Scores for *G. mellonella* injected with varying doses of *N. gonorrhoeae* P9-17. Healthy larva (n = 10 per group) were infected with differing inocula of gonococci, incubated at 37°C and examined with the Health Index Scoring System (HISS) over a period of 48 h. The symbols represent the mean health index score of 3 independent experiments and the error bars represent the standard deviation of the mean. Unpaired t test was done to compare at each time point between gonococcal-infected HISS data and control larvae injected with GC broth alone. * denotes statistically significant difference for all data points with P < 0.05. **C)** Recovery of *N. gonorrhoeae* from *G. mellonella* hemolymph. At least 30 larvae were infected with 10 µl of 8 × 10^7^ CFU *N. gonorrhoeae* P9-17. Survival of *N. gonorrhoeae* within live and dead *G. mellonella* was determined by cutting the end of the larva, extracting hemolymph and plating it on to the VCNT supplemented GC agar plates at different time points (16, 24, 40, and 48 h). Data are pooled from four independent experiments with 1 larva per time-point per experiment. Symbols represent mean survival of larvae and the error bars show standard deviation of the mean. Unpaired t test was done to compare between live and dead CFU from larvae at each time point

In our first experiments, we injected *G. mellonella* larvae with various doses of *N. gonorrhoeae* P9-17 (Pil^+^ Opa_b_^+^) and examined larval survival over 48 h (Figure 1(a)). We observed a dose-dependent relationship with larval survival, with increasing doses of bacteria reducing survival. A suspension of optical density (OD) of 0.6 (~9 × 10^6^ CFU/larva) caused >50% death by 30 h and higher doses of OD 0.8 (~8 × 10^7^ CFU/larva) and 1.0 **(~**6 × 10^8^ CFU/larva) were the most lethal, causing >80% death by 48 h, compared to larvae injected with supplemented GC broth alone (P < 0.001). The mortality observed with the OD 0.8–1.0 doses was significantly greater than the mortality observed with all doses ≤ OD 0.6 (P < 0.05). For all subsequent experiments, we chose a standard inoculum of OD 0.8 (~8 × 10^7^ CFU/larva) as this gave consistent and reproducible killing of >50% of larvae by 18 h.

During these initial infection experiments, we also used a Health Index Scoring System (HISS) to score the health of the larvae. HISS enabled measurement of some of the more subtle differences that were observed during infection (Table 2). Generally, HISS scores declined over time with infection and were dose-dependent (Figure 1b). Compared to larvae injected with control supplemented GC broth, larvae infected with the lowest dose of OD 0.1 (~8.6 × 10^5^ CFU/larva) showed a significant gradual decline in score to ~55 HISS points by 48 h (P < 0.05), whereas the highest doses tested of OD 0.8 − 1.0 (~6.7 × 10^7^ to 6.7 × 10^8^ bacteria/larva) showed significantly reduced HISS scores of ~20 by 48 h (Figure 1b) (P < 0.05).

We also examined the levels of P9-17 within *G. mellonella* over time by sampling larval hemolymph onto selective agar medium plates. Larvae were infected with OD 0.8 (~8 × 10^7^ bacteria/larva) and hemolymph was sampled from both live and recently dead larvae at intervals over 48 h. There was a rapid decrease in larval survival from 100% to 30% by 48 h. We also observed that bacterial numbers gradually fell from mean values of ~10^8^ at 0 h to ~10^2^ CFU/larva in live larvae and to ~10^4^ in dead larvae by 48 h (Figure 1c). There were no significant differences between the numbers of bacteria recovered from live and dead larvae at each time point (P > 0.05).

### *Detailed histopathology examination of* G. mellonella *infected with* N. gonorrhoeae *strain P9-17*

**Figure 2.**
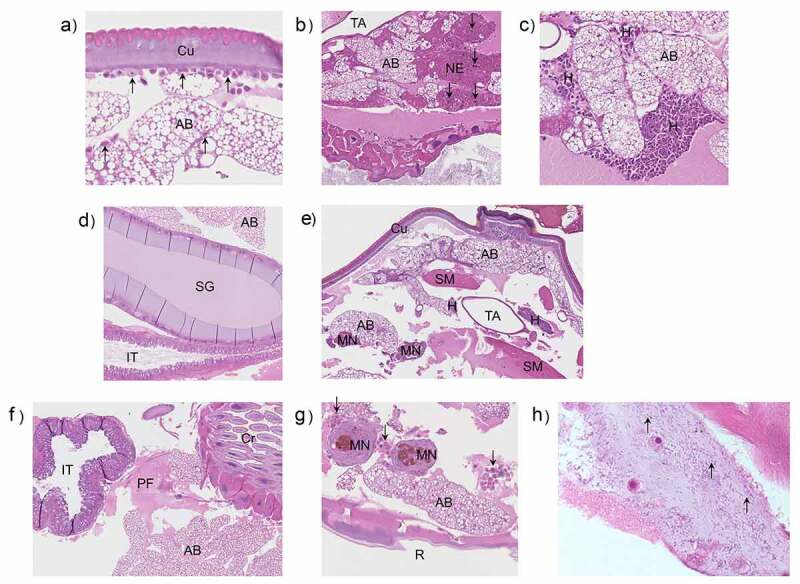
**Histopathology examination of *Galleria mellonella* infected with *Neisseria gonorrhoeae.*** Larvae were infected with *Neisseria gonorrhoeae* P9-17 (OD 0.8, ~8 × 10^7^ CFU/larva) and histopathology examined at 2, 16 and 24 h after infection on fixed larval sections following H&E staining. Uninfected larvae were processed at the same time points. The images are representative of several larvae as described in Table S1. **A)** 2 h post-injection control, larva 1. Normal adipose body and no hemocyte reaction. Hemocytes form a monolayer in the subcuticular space and individual hemocytes are scattered within the adipose body. AB = adipose body, Cu = cuticle, arrows = hemocytes. **B)** 2 h gonococcal infected, larva 4. Locally extensive necrosis of the adipose body with scattered melanin is present between the crop and tracheal apparatus. AB = adipose body, Ne = necrosis, TA = tracheal apparatus, Cr = crop, arrows = melanin. **C)** 2 h gonococcal infected, larva 4. There are large numbers of hemocytes forming a densely cellular aggregate adjacent to the adipose body. Additional hemocytes are filling space between folds of the adipose body. AB = adipose body, H = hemocytes. **D)** 16 h post-injection control, larva 3. Normal adipose body (AB), silk gland (SG), and intestinal tract (IT). **E)** 16 h gonococcal infected, larva 3. Aggregates of hemocytes, occasionally with melanin, are present multi-focally throughout the proleg. AB = adipose body, Cu = cuticle, SM = skeletal muscle, H = hemocytes, MN = melanized nodule, TA = tracheal apparatus. **F)** 24 h post-injection control, larva 3. There is proteinaceous fluid in the coelom adjacent to the crop and intestinal tract. AB = adipose body, Cr = crop, IT = intestinal tract, PF = proteinaceous fluid. **G)** 24 h gonococcal infected, larva 1. Multiple melanized nodules and clusters of hemocytes within the coelom adjacent to the rectum. MN = melanized nodules, AB = adipose body, R = rectum, arrows = hemocyte clusters. **H)** Presence of *N. gonorrhoeae* P9-17 within *G. mellonella* tissue (magnification is x100)

**Figure 3.**
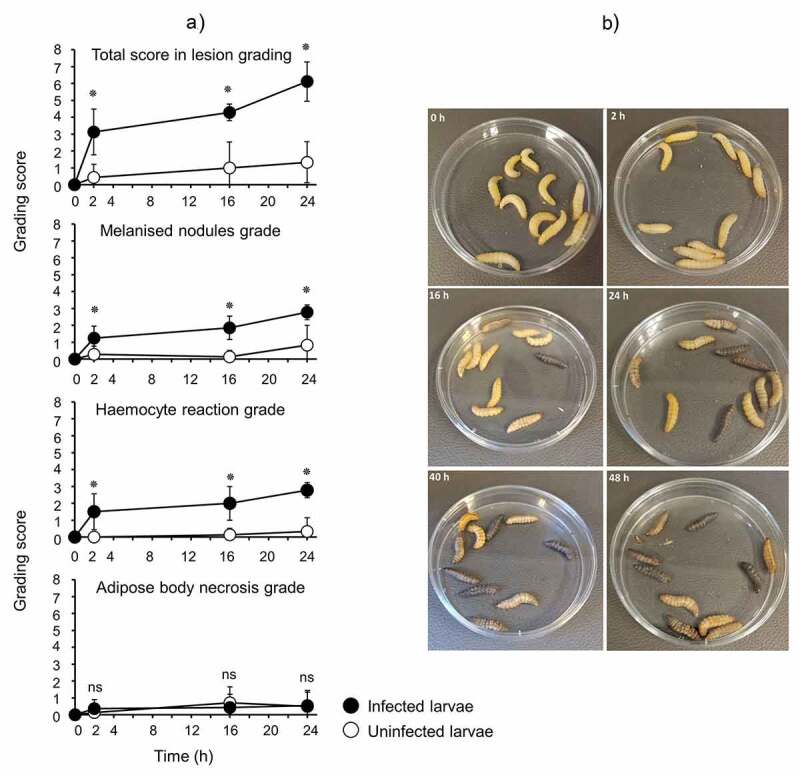
**A) Use of a common lesion grading scheme for *Galleria mellonella* infected with *N. gonorrhoeae* strain P9-17**. Groups of larvae infected with strain P9-17 (OD 0.8, ~8 × 10^7^ CFU/larva) were assessed over 24 h for melanization, adipose body necrosis and hemocyte reaction grades to provide a total score in lesion grading. The symbol represents the mean and error bars the standard deviation of the mean. Unpaired t test was done to compare the scores between infected and uninfected larvae. Ns, not significant (p > 0.05) and * denotes statistical significance, with P < 0.01. **B) Representative images of larvae infected with gonococci showing increasing levels of melanization with time.**

Larvae were infected with OD 0.8 (~8 × 10^7^ CFU/larva) of *N. gonorrhoeae* P9-17 and processed at 2, 16, and 24 h post-infection for H&E staining and histopathology examination. Uninfected larvae were processed at the same time points. Representative H&E stained images are shown in Figure 2. The grading scores for numbers of melanized nodules, adipose body necrosis grade, hemocyte reaction grade and total grade score for individual larva examined are shown in Table S1. Compared to the uninfected control larvae, infection resulted in significant increases in the total lesion grade (P < 0.01), in melanized nodules (P < 0.01) and hemocyte reaction (P < 0.01) at each time point, but there was no significant difference in adipose body necrosis (P > 0.05) (Figure 3(a)). Representative images of increased melanization over time in whole larvae infected with P9-17 are shown in Figure 3b.

### *Infection of* G. mellonella *with phenotypic variants of* Neisseria gonorrhoeae *strain* P9-17 *and other gonococcal isolates*

**Figure 4.**
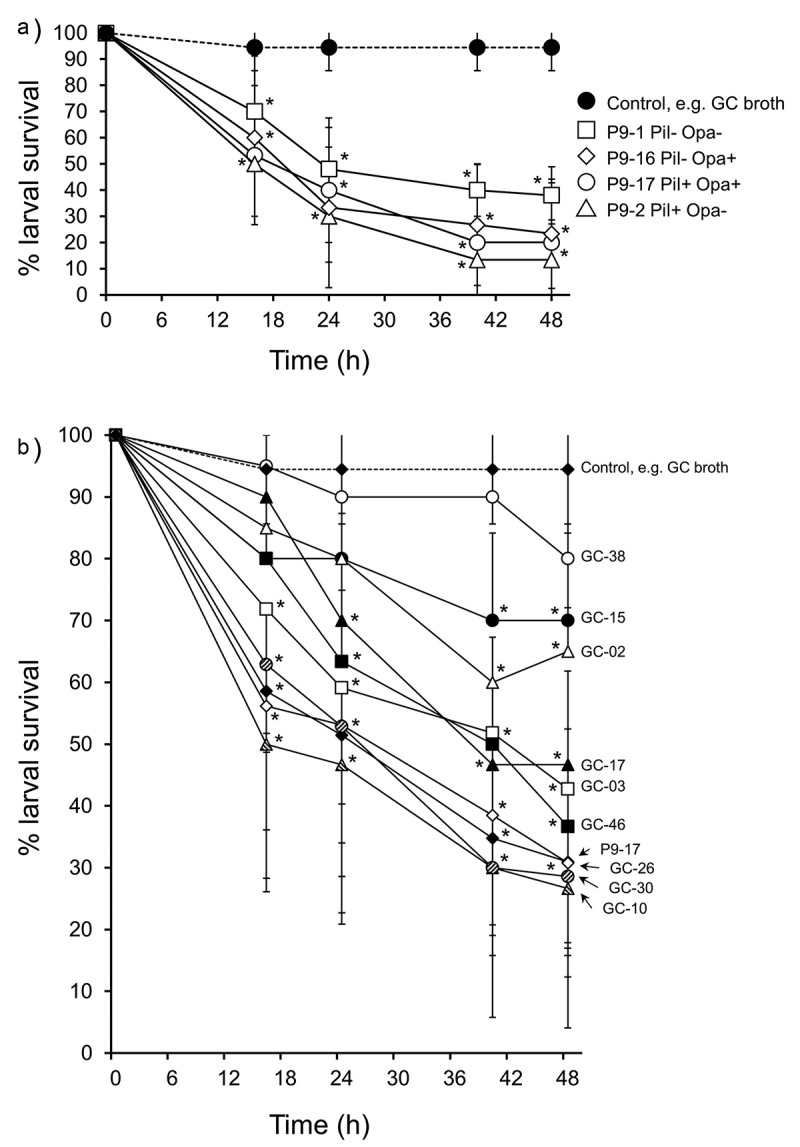
**A) Infection of *Galleria mellonella* with different phenotypic variants of *Neisseria gonorrhoeae* strain P9**. Healthy larva (n = 10 per group) were infected with different P9 variants (~6-8 × 10^7^ CFU per larva of P9-1 Pil- Opa-; P9-2 Pil+ Opa-; P9-16 Pil- Opa+; P9-17 Pil+ Opa_b_+), incubated at 37°C and examined for survival over a period of 48 h. Symbols represent mean survival from a minimum of 3 independent experiments and error bars represent the standard deviation of the mean. Unpaired t test was done at each time point to compare survival of larvae infected with P9 variants and control, GC-injected larvae. * denotes statistical significance with P < 0.01. **B) Infection of *Galleria mellonella* with different *Neisseria gonorrhoeae* isolates**. Healthy larva (n = 10 per group) infected with different gonococcal isolates (~6-8 × 10^7^ CFU per larva), incubated at 37°C and examined for survival over a period of 48 h. Symbols represent the mean survival from a minimum of 3 independent experiments and the error bars represent the standard deviation of the mean. Unpaired t test was done to compare survival of larvae infected with the different isolates and control, uninfected larvae injected with GC broth alone. * denotes statistical significance with P < 0.001. In addition, Unpaired t test was done to compare survival of the larvae infected with the different isolates compared to P9-17 (see text for statistical significance)

The pilus and Opa protein are cardinal molecules mediating gonococcal interactions with host cells [45]. We injected larvae with OD 0.8 (~8 × 10^7^ CFU) of different pilus and Opa protein-expressing phenotypic variants and found that they significantly reduced larval survival to between 10% and 40% by 48 h (P < 0.01), compared with 100% survival of control larvae injected with supplemented GC broth. However, there were no significant differences (P > 0.1) between the phenotypic variants in causing larval death (Figure 4(a)). Sialylation of gonococcal LOS glycans is a virulence mechanism used by gonococci to evade the human immune system [46]. We examined if sialylation of gonococcal LOS increased the virulence of bacteria in *G. mellonella*. Larvae were infected with OD 0.1, 0.6 and 1.0 of untreated and CMP-NANA grown P9-17 and survival monitored over 48 h. As expected, survival was increasingly reduced with doses of OD 0.6 and 1.0 bacteria, compared with control larvae injected with supplemented GC broth alone (P < 0.05). However, there was no statistically significant difference (P > 0.05) in the survival curves of larvae infected with untreated (non-sialylated) and sialylated bacteria (Figure S2).

We also used the model to examine the virulence of other gonococcal isolates, in this case, of several representative isolates from the CDC/FDA AR Panel with reported high Minimum Inhibitory Concentration (MIC) values to ceftriaxone and azithromycin, the antibiotics recommended for treating gonorrhea (Figure 4(b)). Larvae were infected with OD 0.8 (~8 x 10^7^ CFU/larva) of 9 different isolates and also with P9-17, and survival recorded over 48 h. Compared with larvae injected with supplemented GC broth alone, survival of larvae was significantly reduced by 48 h after injection with all of the isolates (P < 0.001) except for GC-38, which did not induce significant death of larvae (P > 0.05). Isolates GC-03, −10, −17, −26, −30, −46 and P9-17 were toxic to larvae, with an average of 30–45% of larvae surviving after 48 h. By contrast, isolates GC-02, −15 and −38 appeared to be less virulent, with an average of ~70-85% of larvae surviving bacterial challenge after 48 h (Figure 4(b)).

### *Killing of* G. mellonella *requires infection with live gonococci*

**Figure 5.**
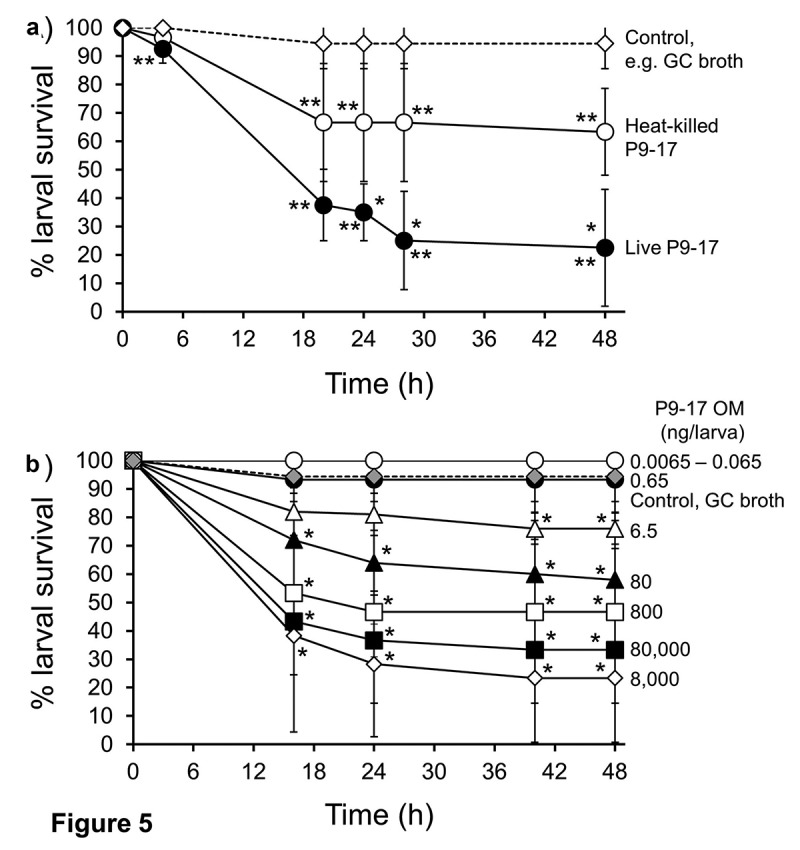
**A) The effect of heat-killed and live inocula of *Neisseria gonorrhoeae* on the survival of *G. mellonella.*** Healthy larva (n = 10 per group) were infected with heat-killed or live *N. gonorrhoeae* strain P9-17 gonococci (~8 × 10^7^ CFU per larva), incubated at 37°C and examined for survival by response to touch over a period of 48 h. Symbols represent the mean survival from a minimum of 3 independent experiments and the error bars represent the standard deviation of the mean. Unpaired t test was done to compare survival between the two groups at all the time points where * denotes statistical significance with P < 0.05. Unpaired t test was also done to compare survival of larvae infected with heat-killed or live bacteria and with control larvae injected with GC broth, where ** denotes statistical significance with P < 0.01. **B) The effect of *Neisseria gonorrhoeae* P9-17 Outer Membranes (OM) on the survival of *Galleria mellonella***. Healthy larva (n = 10 per group) were injected with varying doses of OM (up to 80,000 ng per larva), incubated at 37°C and examined for survival by response to touch over a period of 48 h. Symbols represent the mean survival from a minimum of 3 independent experiments and the error bars represent the standard deviation of the mean. Unpaired t test was done at each time point to compare survival of larvae injected with OM and control group injected with GC broth. * denotes statistical significance with P < 0.01

Larvae were injected with a dose of OD 0.8 (~8 x 10^7^ CFU) live *N. gonorrhoeae* P9-17/larva or with a similar dose of killed (heat-inactivated, HI) bacteria. Survival of larvae was significantly lower after infection with live gonococci compared with HI-bacteria at all the time points (P < 0.05). As expected, survival of larvae infected with live bacteria was ~20% by 48 h (P < 0.01), whereas ~70% of larvae survived after injection of HI-bacteria (P < 0.01) (Figure 5(a), when compared with control larvae injected with supplemented GC broth only.

The release of OM by *N. gonorrhoeae* is a recognized virulence mechanism during infection [47] and we tested the hypothesis that gonococcal OM vesicles could induce larval death. In our first experiments, larvae were injected with *N. gonorrhoeae* P9-17 OM doses from 0.0065 to 6.5 ng/larvae, which would approximate roughly to 6.5 × 10^5^–6.5 × 10^8^ *Neisseria* bacteria, based on LOS content [48,49]. Doses of 0.0065–0.65 ng OM/larva did not significantly affect survival of larvae compared with supplemented GC control injected larvae (P > 0.05). Significant death of ~25% of the larvae was first observed with a dose of 6.5 ng/larva (P < 0.01). Injection of a physiological excess of OM from between 80 and 800 ng OM/larva reduced survival of larvae by ~50% (P < 0.01) by 48 h, and doses of 8000–80,000 ng OM/larva reducing larval survival to <30% (P < 0.01) by 48 h (Figure 5(b)).

## Infection of *G. mellonella* with other *Neisseria spp*

**Figure 6.**
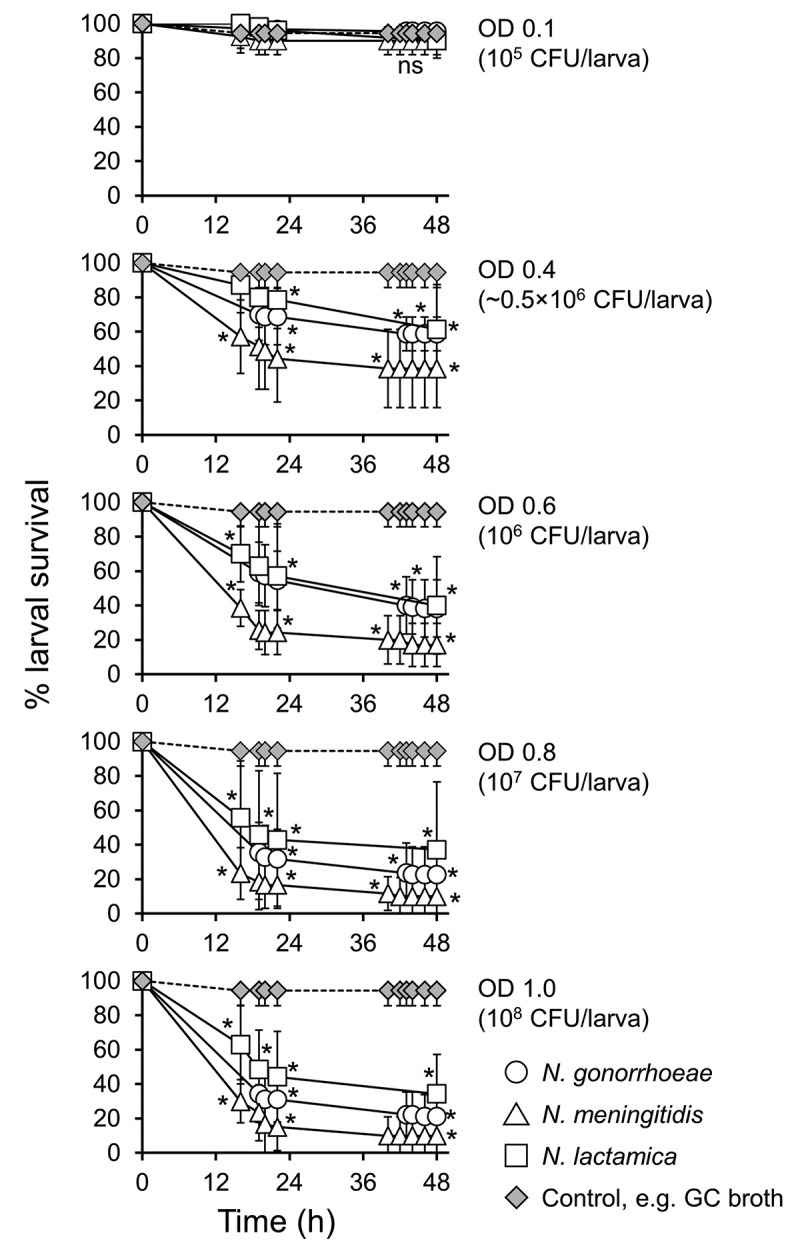
**Survival of *Galleria mellonella* infected with other *Neisseria* species**. Healthy larva (n = 10 per group) were infected with differing doses *N. gonorrhoeae, N. meningitidis*, or *N. lactamica*, incubated at 37°C and examined for survival by response to touch over a period of 48 h. Symbols represent the mean survival from a minimum of 3 independent experiments and error bars represent the standard deviation of the mean. Unpaired t test was done to compare survival of larvae infected with the different *Neisseria spp*. and the control GC broth group at each time point. * denotes statistical significance with P < 0.02. Unpaired t test was also done at each time point to compare between the different *Neisseria spp*. on survival of *G. mellonella*. ns, not significant p > 0.05

We tested the hypothesis that other *Neisseria* species, that is, *N. meningitidis* and *N. lactamica*, could also infect *Galleria mellonella* larvae, but that they would show differences in virulence compared with each other and with gonococci. Groups of larvae were infected with different doses of *N. gonorrhoeae* P9-17, *N. meningitidis* MC58 or *N. lactamica* Y92-1009 and survival recorded over 48 h. At a dose of OD 0.1 (~10^5^ CFU/larva), there was no significant difference in survival of the larvae compared to larvae injected with supplemented GC broth alone (P > 0.05). Increasing doses of OD 0.4–1.0 (~ 0.5 × 10^6^ – ~10^8^ CFU) of all three bacteria decreased larval survival significantly (P < 0.02) (Figure 6). Although there seemed to be a trend of increased virulence of meningococci over both gonococci and *N. lactamica*, it was not statistically significant (P > 0.05). To give an idea of the relative toxicity of the *Neisseriae*, we also tested *P. aeruginosa* PAO-1 in parallel, using doses from 150 CFU/larva up to ~2.5 × 10^5^ CFU/larva (Figure S3). Compared to control larvae injected with supplemented GC broth alone, *P. aeruginosa s*ignificantly reduced larval survival (P < 0.01) with all doses tested, with no survival by 12–18 h in any of the groups.

### *Effects of priming* G. mellonella *larvae with nontoxic doses of* N. gonorrhoeae

**Figure 7.**
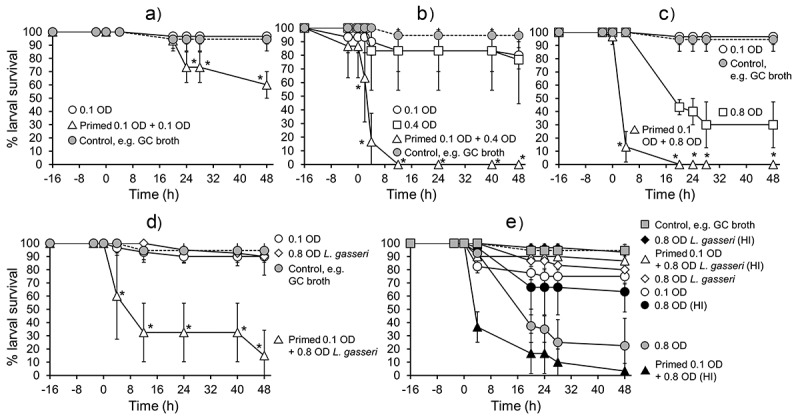
**Effects of priming *Galleria mellonella* larvae with *Neisseria gonorrhoeae***. Healthy larvae (n = 10 per group) were primed by injecting with a non-killing dose of *N. gonorrhoeae* strain P9-17 at −16 h (OD 0.1, ~10^5^ CFU per larva) and then infected at 0 h with **A)** 0.1 OD, ~10^5^ CFU per larva, **B)** 0.4 OD, ~10^6^ CFU per larva, **C)** 0.8 OD, ~10^7^ CFU per larva, or **D)** 0.8 OD ~10^7^ CFU per larva of *L. gasseri*. Controls included infection with OD 0.1 **A)**, 0.4 **B), C)** 0.8 and **D)** 0.8 of bacteria alone from −16 h. Larvae were incubated at 37°C and examined for survival by response to touch over a period of 48 h. Unpaired t test was done to compare survival of larvae in the primed and infected groups with the control group infected with the bacteria alone. * denotes statistical significance with P < 0.05. In **E)**, larvae were primed at −16 h with an OD 0.1 dose of *N. gonorrhoeae* P9-17 and challenged at 0 h with OD 0.8 of heat-inactivated (HI) P9-17 or HI *L. gasseri*. Unpaired t test was done to compare survival of larvae between the different treatments, with statistical significance denoted by P < 0.05 (see text for details). Symbols in each panel represent the mean survival from a minimum of 3 independent experiments and the error bars represent the standard deviation of the mean

In our study, we tested the hypothesis that pre-exposure of *G. mellonella* larvae to nontoxic doses of gonococci protected the larvae from subsequent challenge with various doses of the same organism. Larvae were primed with the nontoxic dose of 0.1 OD (~10^5^ CFU/larvae) *N. gonorrhoeae* P9-17 and after 16 h they were injected with 0.1 OD, 0.4 OD (~8 × 10^6^ CFU/larvae) or 0.8 OD (~8 × 10^7^ CFU/larvae) of P9-17. Larvae were also injected with the higher doses of 0.4 and 0.8 OD as controls. Larval survival was followed for 48 h. As expected, doses of 0.1 and 0.4 OD showed little significant killing of larvae, whereas only ~30% of larvae survived after infection with OD 0.8 by 48 h, compared to larvae injected with supplemented GC broth only (P < 0.05) (Figure 7a- c). When we injected an OD 0.1 dose of P9-17 into primed larvae, larval survival was reduced (P < 0.05) to 60% by 48 h, compared to the non-primed larvae infected with OD 0.1 (Figure 7a). With an OD 0.4 dose, there was a greater rapid decline in survival of primed larvae and they were all dead by 12 h (Figure 7b) (P < 0.05). Even compared to the higher toxic dose of OD 0.8, primed larvae injected with the same dose were more susceptible, with >90% death of the larvae by 4 h and no survivors by 16 h (P < 0.05) (Figure 7c).

We tested next the hypothesis that a heterologous bacterium that is ordinarily nontoxic to *G. mellonella* could be rendered toxic to larvae that had been pre-exposed to gonococci. We examined a trio of *Lactobacillus spp*., which are common commensals of the female genital tract microbiome that may play niche-occupying and protective roles against sexually transmitted infections [50]. We compared *L. brevis, L. crispatus,* and *L. gasseri* and found that both *L. brevis and L. crispatus* significantly reduced larval survival at all doses tested (OD 0.1–1.0, ~10^5^ – 10^8^ CFU/larva) (P < 0.01), compared with larvae injected with supplemented GC broth alone, whereas >80% of all larvae injected with even the highest doses of *L. gasseri* (OD 0.8 − 1.0, ~10^7^ – 10^8^ CFU/larva) survived (P > 0.05) (Figure S4a). Thus, to test the hypothesis, we primed larvae with an OD 0.1 dose of *N. gonorrhoeae* P9-17 and then challenged them with a high dose (OD 0.8, ~10^7^ CFU/larva) of the nontoxic *L. gasseri*. Figure 7d clearly shows that doses of OD 0.1 P9-17 and OD 0.8 *L. gasseri* are not toxic, with >90% survival of larvae by 48 h, compared to larvae injected with supplemented GC broth alone (P > 0.05). By contrast, injection of OD 0.8 *L. gasseri* into gonococcal-primed larvae resulted in rapid death of the larvae, with only 30% surviving by 12 h and ≤10% by 48 h (P < 0.05).

Our experiments described in Figure 5a showed that live gonococci were necessary to kill *G. mellonella* larvae. We tested the hypothesis that injection of HI-gonococci or HI-*L. gasseri* similarly did not kill larvae that had been pre-exposed to gonococci. In these experiments, we primed larvae with a nontoxic OD 0.1 dose (~0.7 × 10^5^ CFU/larva) of *N. gonorrhoeae* P9-17 and challenged them after 16 h with high doses of OD 0.8 of HI-gonococci or HI-*L. gasseri* bacteria (~10^7^ – 10^8^ CFU/larva) (Figure 7e). As expected, when compared to larvae injected with supplemented GC broth alone, ~70-80% of larvae survived after injection of OD 0.1 of live P9-17 or OD 0.8 of HI-gonococci by 48 h (P > 0.05) (Figure 7e), whereas only ~20% of larvae survived after injection of OD 0.8 of live gonococci (P < 0.05). Injection of OD 0.8 of HI-gonococci into larvae pre-exposed to gonococci resulted in significant larval death, with no survivors by 48 h (P < 0.05). In contrast, the injection of OD 0.8 of HI-*L. gasseri* was nontoxic for gonococcal-primed larvae, with >90% larval survival at 48 h (Figure 7e) (P > 0.05).

One possible explanation for our observations is that exposure of larvae to nontoxic doses of gonococci affects the larval innate immune response system, possibly by engaging hemocytes and making them unavailable to control any secondary gonococcal exposure. To test this hypothesis, we injected larvae with various doses of liposomes (Lip) containing dichloromethylene-bisphosphonate (clodronate, CDLip) in order to try and deplete hemocytes, and after 16 h we exposed the larvae to various doses of *N. gonorrhoeae* P9-17 and measured larval survival over 48 h. Lip and CDLip alone showed no significant toxicity toward larvae (<10% death over 64 h) (Figure S5). Larval survival was significantly reduced in gonococcal-infected larvae that had been pre-treated with CDLip, compared with gonococcal-infected larvae pre-treated with Lip. For example, with an OD of 0.1 (~0.7 × 10^5^ CFU/larva), the survival of CDLip-treated larvae by 48 h was reduced by ~20-50% with neat (P = 0.05) and 1/5 dilution (P < 0.02) of CDLip, respectively. With an OD of 0.4 (~8 × 10^6^ CFU/larva), despite the wide error bars, survival of larvae treated with both doses of CDLip was reduced by ~50% by 48 h. Furthermore, even with the highest dose of OD 0.8 (~8 × 10^7^ CFU/larva) gonococci tested, CDLip pre-treated larvae showed significantly lower levels of survival: by 48 h, there was ~40-50% survival amongst Lip-pre-treated larvae, and both neat and 1/5 doses of CDLip reduced survival significantly to <10% by 48 h (P < 0.05) (Figure S5).

### G. mellonella *as a model to examine the efficacy of antimicrobials to treat* N. gonorrhoeae *infection*

There is a large and increasing literature describing the use of the *G. mellonella* model for testing antibiotics [13,31] and we tested the hypothesis that it would be useful for testing the *in vivo* efficacy of the clinically recommended antibiotics ceftriaxone and azithromycin. In preliminary experiments, we observed that neither ceftriaxone nor azithromycin had any significant effect on survival of larvae (P > 0.05), even when tested at doses as high as ~1000 μg/mL (i.e., 10 μg/larva) (Figure S6).

**Figure 8.**
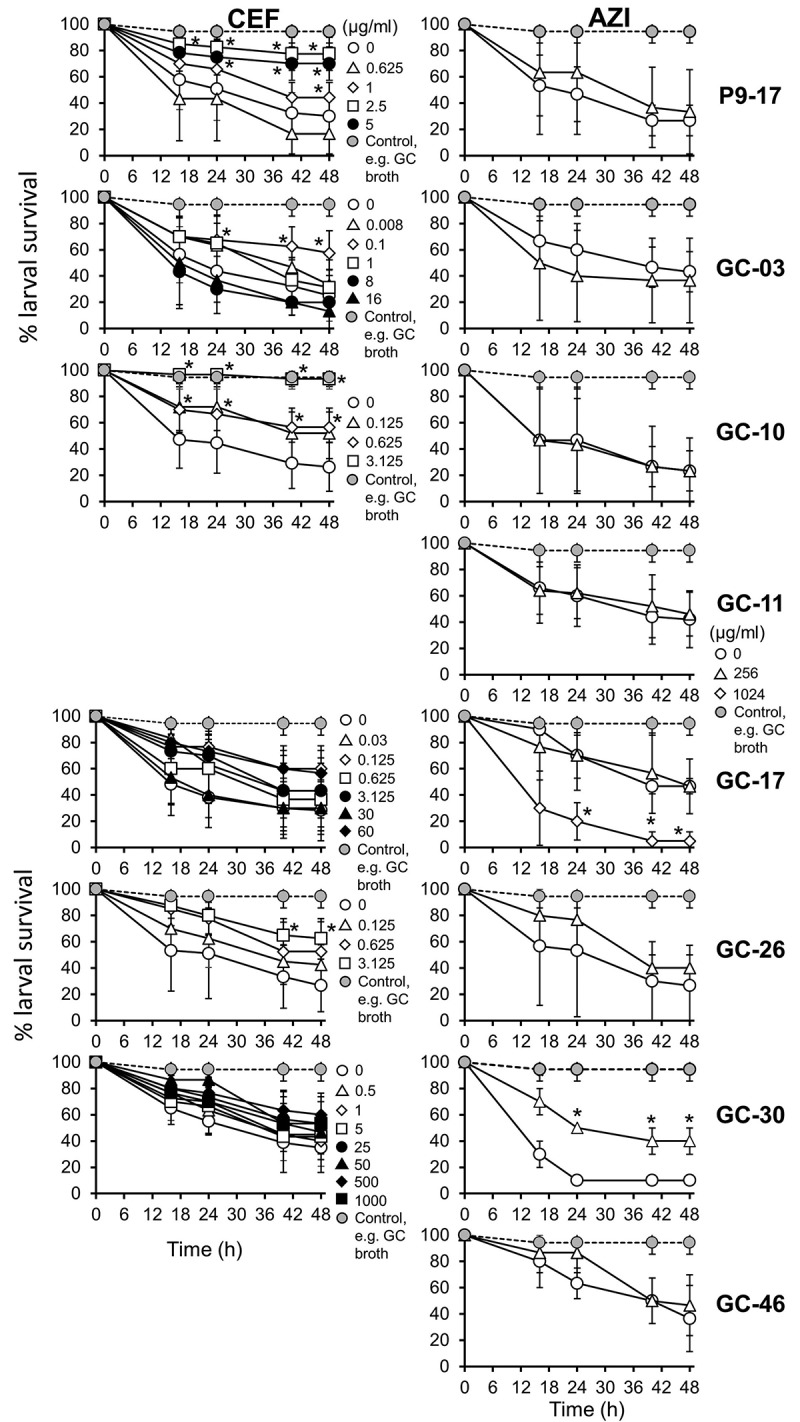
**Effects of treating *Neisseria gonorrhoeae*-infected *Galleria mellonella* with antibiotics ceftriaxone and azithromycin**. Healthy larva (n = 10 per group) were infected with different gonococcal isolates (OD 0.8, ~10^8^ CFU per larva) and injected simultaneously with different doses of ceftriazone or azithromycin antibiotic, incubated at 37°C and examined for survival by response to touch over a period of 48 h. The symbols represent mean survival from a minimum of 3 independent experiments and the error bars represent the standard deviation of the mean. Unpaired t test was done at each time point to compare survival of gonococcal-infected larvae without antibiotic and the survival of gonococcal-infected larvae treated with antibiotic. * denotes statistical significance with P < 0.05

We next infected groups of larvae with different gonococcal strains from the CDC/FDA AR Panel (OD 0.8, ~8 x 10^7^ CFU/larva) that showed high levels of resistance (Table 4) to ceftriaxone and azithromycin and at the same time we administered various doses of the antibiotics alone, or together, to test their efficacy in reducing death. The antibiotic doses chosen reflected the MIC values and higher doses if the MIC values were ineffective. Time course experiments showed that ~30% of larvae survived infection with any of the gonococcal strains by 48 h in the no-antibiotic controls, compared to larvae injected with supplemented GC broth alone (P < 0.05) (Figure 8). There was variation in the ability of ceftriaxone to improve survival in larvae infected with the different isolates, compared to untreated and infected larvae. Doses of >1 μg/mL, >0.625 μg/mL and 3.125 μg/mL of ceftriaxone significantly increased survival of P9-17-, GC-10- and GC-26-infected larvae by an average of 40–60%, respectively, by 48 h (P < 0.05). With isolate GC-03, a low dose of 0.1 μg/mL of ceftriaxone improved survival of larvae by ~40% (P < 0.03), but increasing doses were contra-indicated. However, ceftriaxone did not improve survival of larvae infected with either GC-17 or GC-30, even tested at doses as high as 60 μg/mL for the former and 1000 μg/mL for the latter (P > 0.05) (Figure 8).

**Table 4.** Summary of antibiotic resistance profiles of gonococcal isolates used in this study

Isolate	Ceftriaxone MIC (μg/mL)	Ceftriaxone interpretation	Azithromycin MIC (μg/mL)	Azithromycin interpretation
P9-17	0.15	S	2	S
GC-30	0.125	S	0.5	S
GC-10	0.125	S	1	S
GC-26	0.125	S	1	S
GC-46	0.06	S	0.25	S
GC-17	0.03	S	256	NS
GC-11	0.008	S	16	NS
GC-3	0.008	S	8	NS

Minimum inhibitory concentrations (MIC) and interpretations of that result are shown for azithromycin and ceftriaxone. S stands for susceptible and NS stands for non-susceptible. GC isolates are from the CDC/FDA Antibiotic Resistance Panel (https://wwwn.cdc.gov/ARIsolateBank/Panel/PanelDetail?ID=11)

Treatment of larvae with a high dose of azithromycin (256 μg/mL) did not increase the percentage of survivors amongst larvae infected with OD 0.8 of gonococcal strains P9-17, GC-03, GC-10, GC-11, GC-17, GC-26, or GC-46 (Figure 8). However, azithromycin showed some efficacy in larvae infected with strain GC-30, with larval survival improved by ~30% (P < 0.05). Interestingly, a higher dose of 1024 μg/mL reduced larval survival significantly (P < 0.05) in the presence of isolate GC-17, suggesting a toxic synergism (Figure 8). This higher dose was not tested with the other isolates.

Finally, we tested the efficacy of dual treatment with ceftriaxone and azithromycin in reducing death in larvae that had been infected with OD 0.8 of strains P9-17, GC10, GC-26, or GC-30 (Figure S7). There was a suggestion of synergism between ceftriaxone and azithromycin, but this was isolate-specific and only apparent with low doses of ceftriaxone used. For example, a dose of 0.625 μg/mL ceftriaxone did not protect larvae from killing by P9-17, but addition of azithromycin significantly improved survival by ~70%, despite azithromycin alone having no effect. Similarly, with isolate GC-10, survival of larvae treated with 0.625 μg/mL of ceftriaxone was increased by ~25% when the same dose was administered with azithromycin. For both isolates, where larval survival was improved with increased doses of ceftriaxone, the presence of azithromycin had no significant effect (P > 0.05). By contrast, administration of azithromycin with ceftriaxone to treat larvae infected with isolates GC-26 and GC-30 had no significant effect (P > 0.05). Table 5 summarizes the antibiotic testing data for Figure 8 and Figure S7.

**Table 5.** **Summary of the antibiotic testing data shown in** Figure 8 **and** Figure S7

A) Ceftriaxone (Figure 8)															
**Gonococcal Isolate**															
**P9-17**		**GC-03**		**GC-10**		**GC-17**		**GC-26**		**GC-30**					
**Dose**	**%**	**Dose**	**%**	**Dose**	**%**	**Dose**	**%**	**Dose**	**%**	**Dose**	**%**				
**(µg/mL)**	**LS**	**(µg/mL)**	**LS**	**(µg/mL)**	**LS**	**(µg/mL)**	**LS**	**(µg/mL)**	**LS**	**(µg/mL)**	**LS**				
0	30	0	25	0	26	0	28	0	27	0	35				
0.625	17	0.008	33	0.125	52*	0.03	43	0.125	43	0.5	45				
1	44*	0.1	58*	0.625	57*	0.125	60	0.625	53	1	40				
2.5	78*	8	20	3.125	93*	0.625	37	3.125	63*	5	43				
5	70*	16	13			3.125	43			25	53				
						30	30			50	47				
						60	57			500	60*				
										1000	53				
B) Azithromycin (Figure 8)															
**Gonococcal Isolate**															
**P9-17**		**GC-03**		**GC-10**		**GC-11**		**GC-17**		**GC-26**		**GC-30**		**GC-46**	
**Dose**	**%**	**Dose**	**%**	**Dose**	**%**	**Dose**	**%**	**Dose**	**%**	**Dose**	**%**	**Dose**	**%**	**Dose**	**%**
**(µg/mL)**	**LS**	**(µg/mL)**	**LS**	**(µg/mL)**	**LS**	**(µg/mL)**	**LS**	**(µg/mL)**	**LS**	**(µg/mL)**	**LS**	**(µg/mL)**	**LS**	**(µg/mL)**	**LS**
0	27	0	43	0	23	0	42	0	47	0	27	0	10	0	37
256	33	256	37	256	23	256	46	256	47	256	40	256	40	256	47*
								1024	5*						
**C) Ceftriaxone (different concentrations) and azithromycin (256 µg/mL)** (Figure S7)															
**Gonococcal Isolate**															
**P9-17**		**GC-10**		**GC-26**		**GC-30**									
**Dose**	**%**	**Dose**	**%**	**Dose**	**%**	**Dose**	**%**								
**(µg/mL)**	**LS**	**(µg/mL)**	**LS**	**(µg/mL)**	**LS**	**(µg/mL)**	**LS**								
0	30	0	26	0	27	0	28								
0.625	83*	0.625	73*	0.625	48	0.625	10								
3.125	70*	3.125	80*	3.125	60*	3.125	17								

Summary of data for *G. mellonella* % larval survival (%LS) after 48 h. Larvae were injected with different *N. gonorrhoeae* isolates and treated with varying doses of ceftriaxone, azithromycin, or dual treatment with one concentration of azithromycin and various concentrations of ceftriaxone. * indicates significant difference by Unpaired T test between control group (0 µg/mL) and test group.

There is also an increasing literature describing the use of the *G. mellonella* model to test the bioactivity of novel antimicrobials, for example, manganese(i) tricarbonyl complexes, theaflavin-epicatechin combinations, and carbon monoxide-releasing molecule [Mn(CO)_3_(tpa-kappa_3_N)]Br, to cite a few [51–53]. We used the model to examine whether two novel antimicrobial compounds, monocaprin [54] and 5-Mercapto-2-nitrobenzoic Acid-Coated Silver Nanoclusters (MNBA-AgNCs) [42] could stop systemic gonococcal infection *in vivo*. In the current study, we infected *G. mellonella* with 0.8 OD (~8 × 10^7^ CFU/larva) of *N. gonorrhoeae* P9-17 and then treated larvae with various doses of the compounds. Neither monocaprin (0.1–50 mM doses) nor MNBA-AgNCs (0.467–58.44 µM doses) improved the survival of infected larvae (Figure S8), compared to untreated, infected larvae (P > 0.05). In addition, compared to larvae injected with supplemented GC broth alone, monocaprin was significantly toxic at the highest dose of 50 mM tested, reducing larval survival by 80% (P < 0.05). By contrast, the MNBA-AgNCs showed no toxicity (P > 0.05) (Figure S8).

## Discussion

The key findings from this initial descriptive study are that *N. gonorrhoeae* can infect and kill *G. mellonella* larvae and that increasing gonococcal toxicity correlated with reduced health index scores and pronounced histopathological changes such as increases in the total lesion grade, melanized nodules, hemocyte reaction, and multifocal adipose body degeneration. The cytopathic effect required injection of live bacteria and could be abrogated by heat-killing; moreover, killing could be induced by isolated LOS-replete OM, but only when used at physiologically excess amounts. Larval death was also independent of the expression of pilus or Opa protein and of sialylation of LOS glycans, within a single gonococcal species studied. In the current study, we only used a single dose of ~8 × 10^7^ CFU/larva to compare the different pilus and Opa variants, and future studies could test other doses, which may potentially highlight some differences in pathology, and also the contribution of other possible adhesins. However, the model could demonstrate relative toxicity of different isolates; the majority of the isolates we tested showed comparable toxicity as P9-17, but we also identified isolates that showed reduced toxicity and one that was avirulent. The explanation for this observed variability in toxicity is not clear and may be related to other phenotypic differences than pilus or Opa expression, or growth rate.

There was no difference between gonococci and other *Neisseria spp*., that is, meningococci and lactamica, in their ability to kill the larvae. By contrast, *P. aeruginosa*, which has been exhaustively studied in the larval model, was significantly more cytotoxic to the larvae than gonococci. This is unsurprising, as *P. aeruginosa* possesses an arsenal of virulence factors and toxins [55] and displays a faster growth rate than gonococci. Surprisingly, we found that *L. brevis* and *L. crispatus* killed larvae like gonococci, whereas *L. gasseri* did not. The reasons for this discrepancy are unclear, but could be related to variable production of lactic acid, which is known to kill *G. mellonella* larvae, and/or the differing cell surface architecture, expression and/or secretion of other compounds by these different *Lactobacillus spp* [56].

In simple co-infection experiments in our study, we found that *L. gasseri* failed to improve the survival of *G. mellonella* infected with P9-17 (Figure S4b). However, several studies have reported that *Lactobacillus spp*. can inhibit gonococcal adherence to epithelial cells *in vitro* [57–59] and that the generation of an acidic environment of pH <4.0 associated with *Lactobacillus* metabolism was a major mechanism for counter-acting gonococcal growth [60]. Other mechanisms may include the production of surfactants [60], and bacteriocin-like substances [61] and acidic compounds that have been reported to inhibit GC viability *in vitro* [62] and in a porcine vagina mucosa model [63], respectively. In light of these reported biological effects of lactobacilli on gonococci, further studies could include pH adjustment and the use of other *Lactobacillus spp.*

Priming injection of low doses of microorganisms has been reported in several studies to apparently protect *G. mellonella* larvae from subsequent repeated exposure to the same microorganism or other organisms [35,37,38,64–68]. Conversely, in our study, gonococcal-primed larvae were more susceptible to the pathogenic effects of secondary exposure to both live gonococci and killed gonococci. Moreover, the effect was not organism-specific, and nontoxic *L. gasseri* could kill gonococcal-primed larvae, but only when injected as live organisms and not killed organisms. A possible explanation for the observed difference in the effects of killed gonococci and killed *L. gasseri* is that that gonococci contain heat-stable components that are toxic to hemocyte-depleted larvae. It is also possible that the priming event engages the innate response of the resident larval population of hemocytes, which are then unavailable to potentially control subsequent infection. Many studies have demonstrated the binding of bacterial pathogens to larval hemocytes and their subsequent uptake, for example recent studies with *Yersinia pseudotuberculosis* [69], *Salmonella* Enteritidis [70] and *Coxiella burnetti* [71]. In preliminary confocal experiments we did see an association of gonococci with hemocytes in extracted hemolymph (data not shown), but more detailed studies are necessary to examine these bacterial-larval innate cell interactions, and are outside the scope of the current manuscript.

In our study, larvae were used to examine the efficacy of the clinically used antibiotics ceftriaxone and azithromycin to improve survival of gonococcal-infected larvae. We did not measure bacterial burden within the larvae as the surrogate measure of antimicrobial efficacy, as the numbers of recovered gonococci generally declined over time in untreated larvae, which may have made it more difficult to demonstrate statistically significant antimicrobial activity. Ceftriaxone significantly protected larvae from gonococcal infection. This protection was dose-dependent and varied between the isolates tested, and doses higher than the reported *in vitro* MIC values were necessary for a biological effect *in vivo*. Conversely, azithromycin was ineffective, and even toxic to the larvae when administered in high doses. Previously, we showed that two novel antimicrobial compounds, monocaprin [54] and MNBA-AgNCs [42], were both highly efficient in killing gonococci *in vitro*. Both compounds were developed as topical antimicrobials to treat gonococcal infections and showed no efficacy systemically in preventing gonococcal-induced larval death. Interestingly, a recent study has reported that a different silver nanoparticle preparation that did not carry a bioactive ligand, such as MNBA, could increase the survival of larvae infected with *P. aeruginosa* [72]. The larval model therefore seems to be useful for comparing nanoparticle compounds, and disparities in bioactivity may reflect different mechanisms of action *in vivo*. Furthermore, the larval model in our study did provide an indication of the toxicity of the novel compounds *in vivo*.

The *Galleria* model has advantages over other models for testing the efficacy and toxicity of novel antimicrobials [13]. In drug development, novel antimicrobials are screened initially *in vitro* to determine MICs against pathogens and their toxicity is evaluated in cultured mammalian cell lines. Successful compounds then usually progress to animal infection models to examine *in vivo* efficacy and also to examine pharmokinetic and pharmocodynamic properties. Although necessary, gonococcal, and other pathogen-testing animal models are expensive, time-consuming, and challenging for screening large numbers of compounds that initially are successful in *in vitro* studies. By contrast, the *Galleria* model is a simple, cheap, and highly reproducible alternative for rapid *in vivo* pre-screening of antimicrobial compounds, and offers a bridge between the *in vitro* studies and final animal testing. Potentially, the model could identify and exclude compounds that are either ineffective *in vivo* despite promising MIC determinations and/or show toxicity, and thus reduce the number of compounds that go forward to testing in animal infection models. Thus, pre-screening using the *Galleria* model could lead to a reduction in animal use and supports the 3Rs principles of Replacement, Reduction, and Refinement.

In summary, we have shown that *N. gonorrhoeae* can infect *Galleria mellonella* larvae and induce pathological changes that result in larval death. However, the model has some clear limitations. Such larvae are not a natural target for infection with *Neisseria spp*. Indeed, gonococci did not appear to be multiplying within larvae and CFU numbers gradually declined over time in the hemolymph, although future studies would also examine larval feces for the presence of gonococci. The most probable explanation is that the larvae may not express the specific receptors that make them significantly labile to gonococcal interactions, for example, the presence of pilus-binding receptors, or the presence of CEACAM or heparin sulfate proteoglycan (HSPG) receptors for Opa [73,74]. However, insects are known to produce HSPG molecules [75] and mucopolysaccharides have been reported in *Galleria* [76], and further studies would be needed to examine whether these molecules are involved with binding Opa proteins. In addition, the target receptor of the Opa_b_ variant expressed by strain P9-17 is unknown and would need to be determined. Another possible explanation is that *Galleria* do not possess the specific innate immune responses that can recognize low levels of Pathogen Associated Molecular Patterns (PAMPs) such as *Neisseria* LOS that are indicative of the start of an infectious process. The exact mechanism of inducing larval death is not known, but may involve other toxic components of the gonococcus, for example, the potential release of peptidoglycan fragments or other potential toxins [77,78]. The model may therefore not prove useful for studying gonococcal adhesion and invasion events *in vivo*, but may provide a method for assessing putative toxins/virulence factors, without resorting to cell cultures and mammalian models.

We also attempted to develop the model as an *in vivo* bactericidal assay, by co-injecting live bacteria, antibodies to OM with demonstrable bactericidal activity *in vitro* [79] and human serum complement, and then examining survival over time (data not shown). This proved unviable as a strategy due to the limitations of the volume of sample that can be injected into the larvae without causing swelling and stress. Furthermore, the doses of gonococci required to induce pathology are high, compared with other pathogens. Further possible limitations of the study are the provenance of the waxmoth larvae, whether they have residual antibiotics or whether other commensal bacteria might interfere: thus, future mechanistic studies would make use of untreated larvae with known flora and these are becoming increasingly available from scientific suppliers, albeit at considerably higher cost. Regardless, our expectation for the long-term applicability of the *G. mellonella* larval model is that it may prove most useful as part of the drug discovery pipeline for *in vivo* screening of novel antimicrobials for bioactivity against gonococci and for toxicity, prior to studies in mammalian models.

## Supplementary Material

Supplemental Material

## Data Availability

Data generated for this study have been deposited with the University of Southampton, 10.5258/SOTON/D1818.
