# Supplementary material for: Establishing an invertebrate Galleria mellonella greater wax moth larval model of Neisseria gonorrhoeae infection
Source: Virulence. 2021 Jul 25;12(1):1900–20. doi: 10.1080/21505594.2021.1950269 (PMC8312596; doi:10.1080/21505594.2021.1950269)
Supplement: Supplemental Material [file KVIR_A_1950269_SM6600.zip › supplementary/Supplementary Table1.docx]

**Supplementary Table 1. Histopathology grading of *Galleria mellonella* larvae infected with *N. gonorrhoeae* strain P9-17 – raw data counts.**

| **Time (h) and slide number** | **Number of melanized nodules (grade)** | **Adipose body necrosis grade** | **Haemocyte reaction grade** | **Total grade score** |
| --- | --- | --- | --- | --- |
| 2h control 1 | 0 (0) | 0 | 0 | 0 |
| 2h control 2 | 0 (0) | 0 | 0 | 0 |
| 2h control 3 | 0 (0) | 0 | 0 | 0 |
| 2h control 4 | 7 (1) | 0 | 0 | 1 |
| 2h control 5 | 2 (1) | 1 | 0 | 2 |
| 2h control 6 | 0 (0) | 0 | 0 | 0 |
| 2h control 7 | 0 (0) | 0 | 0 | 0 |
| 16h control 1 | 4 (1) | 2 | 1 | 4 |
| 16h control 2 | 0 (0) | 1 | 0 | 1 |
| 16h control 3 | 0 (0) | 2 | 0 | 2 |
| 16h control 4 | 0 (0) | 0 | 0 | 0 |
| 16h control 5 | 0 (0) | 0 | 0 | 0 |
| 16h control 6 | 0 (0) | 0 | 0 | 0 |
| 16h control 7 | 0 (0) | 0 | 0 | 0 |
| 24h control 1 | 1 (1) | 0 | 0 | 1 |
| 24h control 2 | 7 (1) | 2 | 0 | 3 |
| 24h control 3 | 0 (0) | 0 | 0 | 0 |
| 24h control 4 | 0 (0) | 0 | 2 | 2 |
| 24h control 5 | 3 (1) | 1 | 0 | 2 |
| 24h control 6 | 0 (0) | 0 | 0 | 0 |
|  |  |  |  |  |
| 2h infected 1 | 11 (2) | 0 | 2 | 4 |
| 2h infected 2 | 8 (2) | 0 | 2 | 4 |
| 2h infected 3 | 7 (1) | 0 | 0 | 1 |
| 2h infected 4 | 4 (1) | 1 | 3 | 5 |
| 2h infected 5 | 5 (1) | 0 | 1 | 2 |
| 2h infected 6 | 0 (0) | 0 | 2 | 2 |
| 2h infected 7 | 9 (2) | 1 | 0 | 3 |
| 2h infected 8 | 7 (1) | 1 | 2 | 4 |
| 16h infected 1 | 9 (2) | 0 | 2 | 4 |
| 16h infected 2 | 11 (2) | 0 | 2 | 4 |
| 16h infected 3 | 8 (2) | 0 | 3 | 5 |
| 16h infected 4 | 21 (3) | 2 | 0 | 5 |
| 16h infected 5 | 7 (1) | 1 | 2 | 4 |
| 16h infected 6 | 13 (2) | 0 | 2 | 4 |
| 16h infected 7 | 4 (1) | 0 | 3 | 4 |
| 24h infected 1 | 24 (3) | 0 | 3 | 6 |
| 24h infected 2 | 18 (3) | 0 | 3 | 6 |
| 24h infected 3 | 31 (3) | 2 | 3 | 8 |
| 24h infected 4 | 21 (3) | 0 | 2 | 5 |
| 24h infected 5 | 15 (3) | 2 | 3 | 8 |
| 24h infected 6 | 13 (2) | 0 | 3 | 5 |
| 24h infected 7 | 21 (3) | 0 | 2 | 5 |
| 24h infected 8 | 21 (3) | 0 | 3 | 6 |
| 24h infected 9 | 12 (2) | 1 | 3 | 6 |

The grading scheme used to score the common lesions for larvae is as follows:

**Melanization:**

0 = no melanized nodules

1 = Melanized nodules occupying less than 10 % of the coelomic cavity

2 = Melanized nodules occupying 10-20 % of the coelomic cavity

3 = Melanized nodules occupying > 20 % of the coelomic cavity

**Adipose body necrosis:**

0 = no necrosis

1 = Focal or multifocal necrosis affecting less than 10 % of the tissue

2 = Focal or multifocal necrosis affecting 10-20 % of the tissue

3 = Focal or multifocal necrosis affecting > 20 % of the tissue

**Haemocyte reaction:**

0 = Low numbers of individual haemocytes within the coelom, monolayer lining subcuticular space (normal)

1 = Increased numbers of individual haemocytes, no distinctive clusters

2 = Formation of discrete haemocyte clusters in the subcuticular space and/or coelom

3 = Haemocyte clusters filling the subcuticular space and/or space between coelomic organs
