# Supplementary figures and images for: Establishing an invertebrate Galleria mellonella greater wax moth larval model of Neisseria gonorrhoeae infection
Source: Virulence. 2021 Jul 25;12(1):1900–20. doi: 10.1080/21505594.2021.1950269 (PMC8312596; doi:10.1080/21505594.2021.1950269)

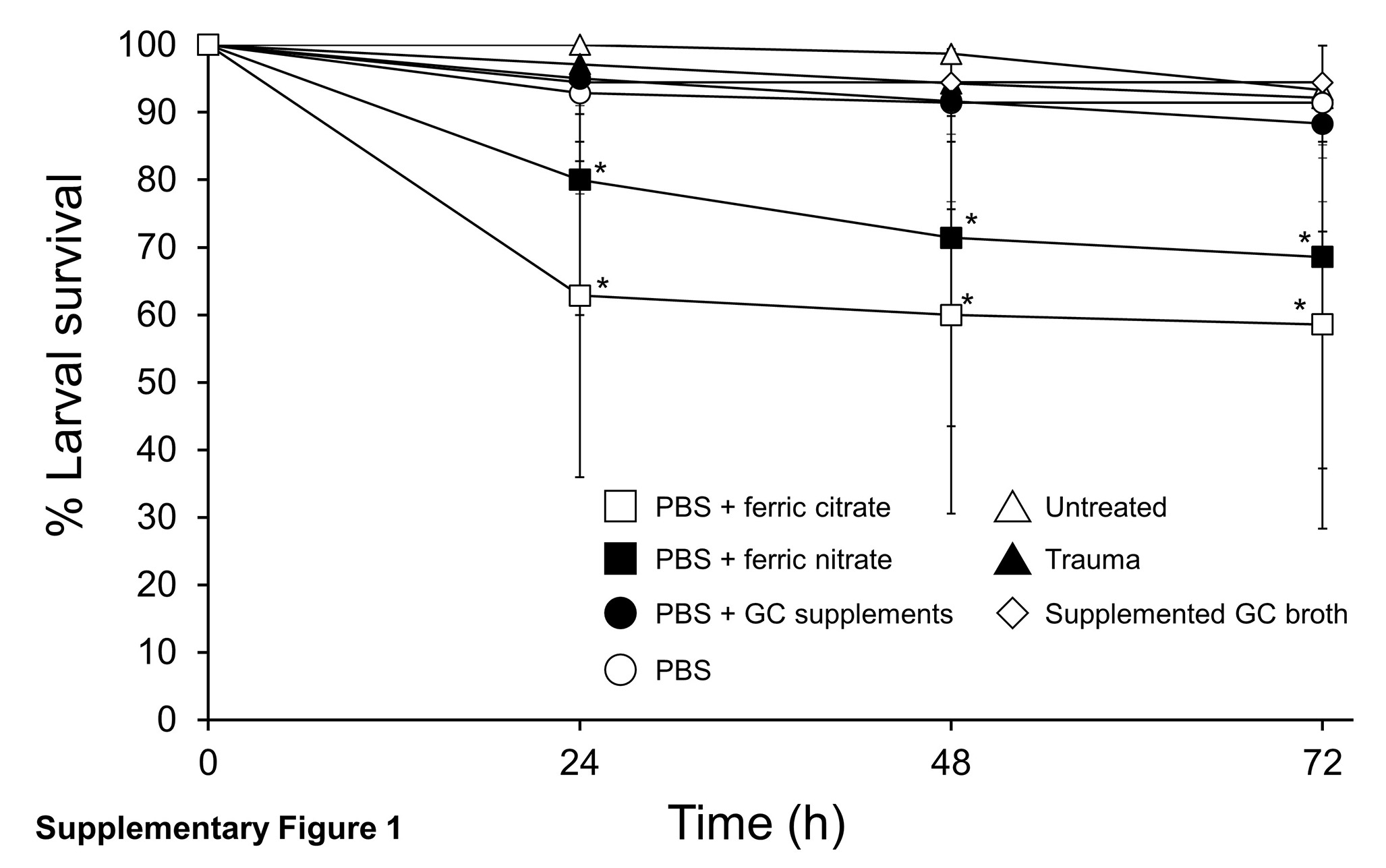

Supplement: Supplemental Material [file KVIR_A_1950269_SM6600.zip › supplementary/Supplementary_Figure1.jpg]

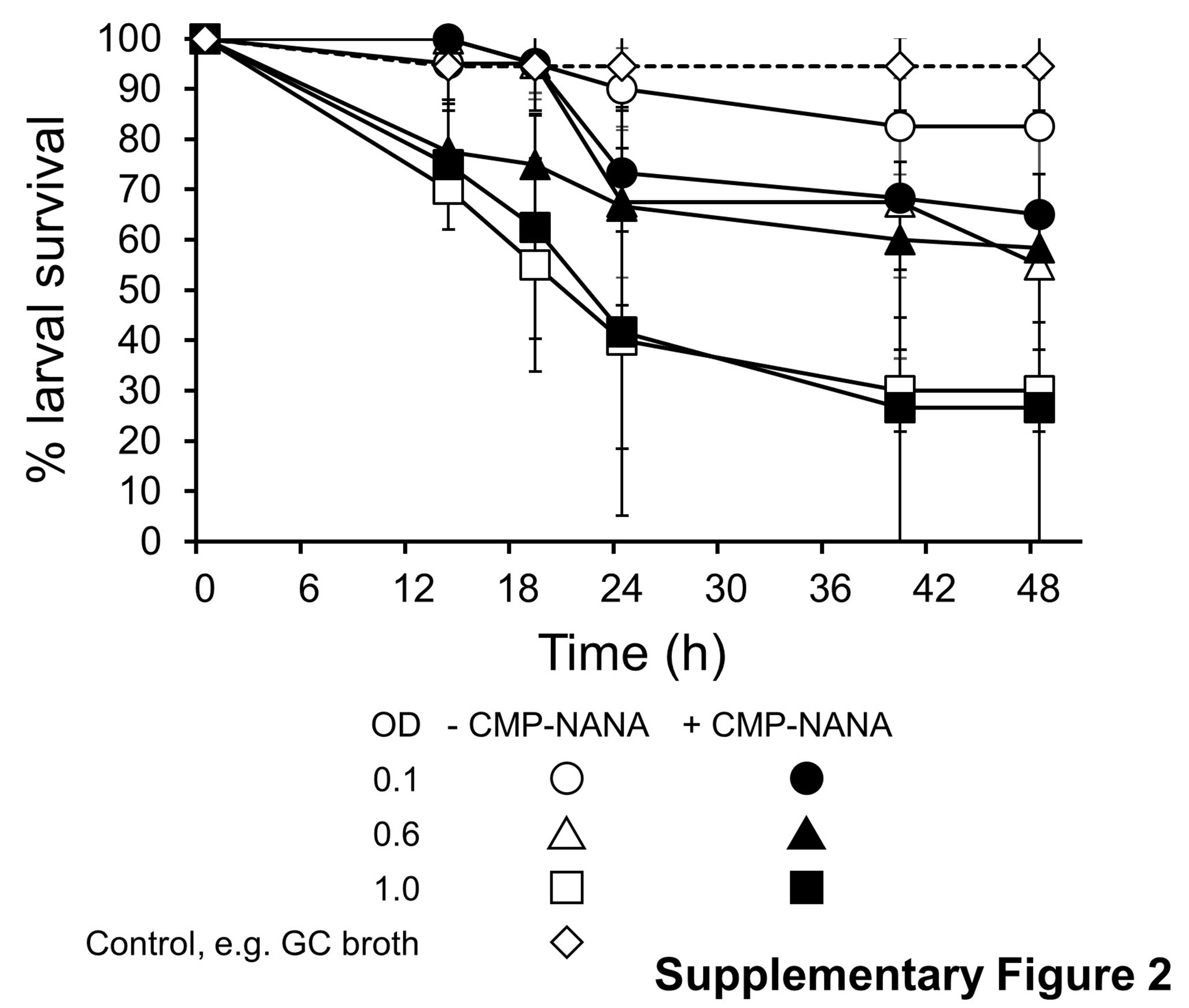

Supplement: Supplemental Material [file KVIR_A_1950269_SM6600.zip › supplementary/Supplementary_Figure2.jpg]

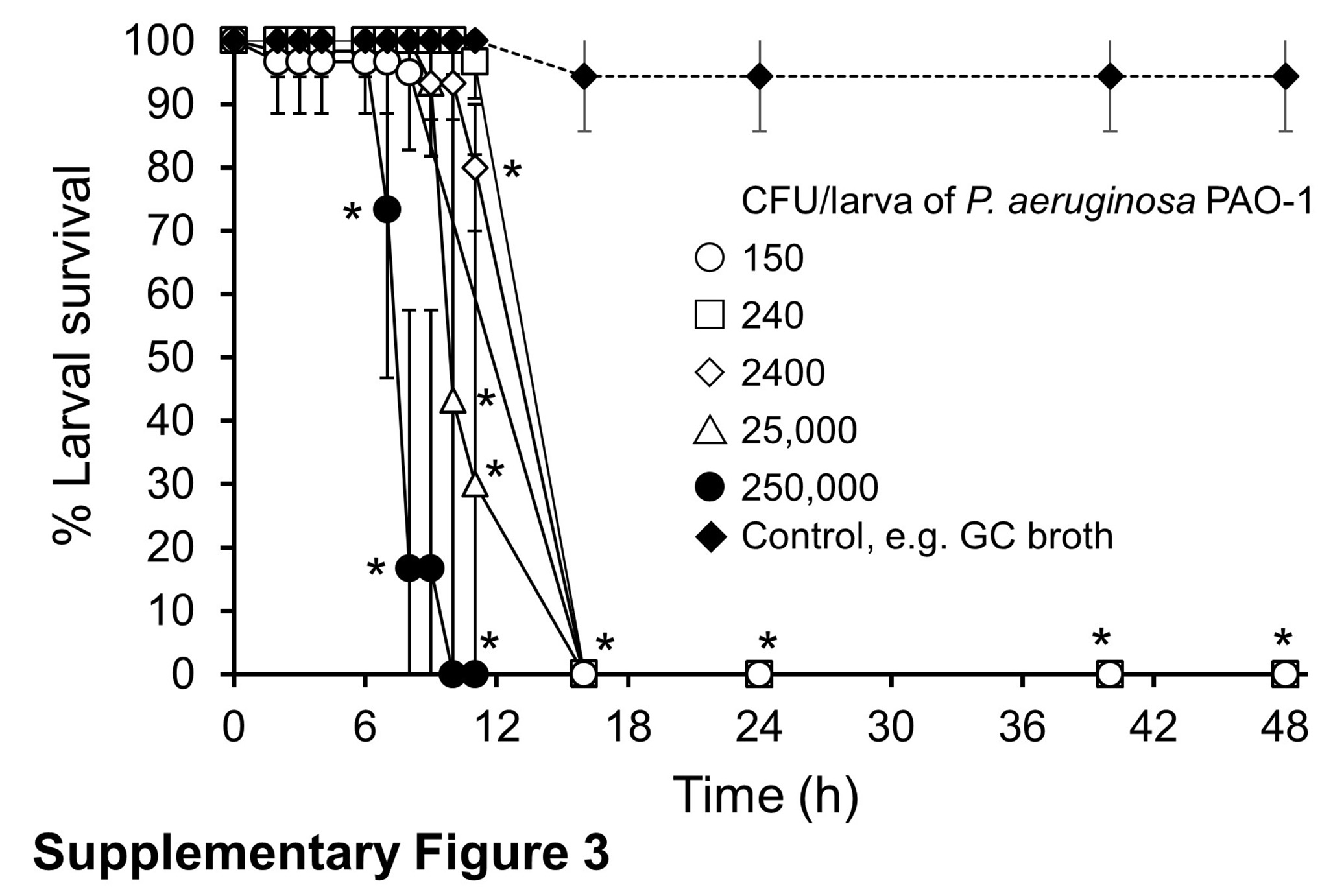

Supplement: Supplemental Material [file KVIR_A_1950269_SM6600.zip › supplementary/Supplementary_Figure3.jpg]

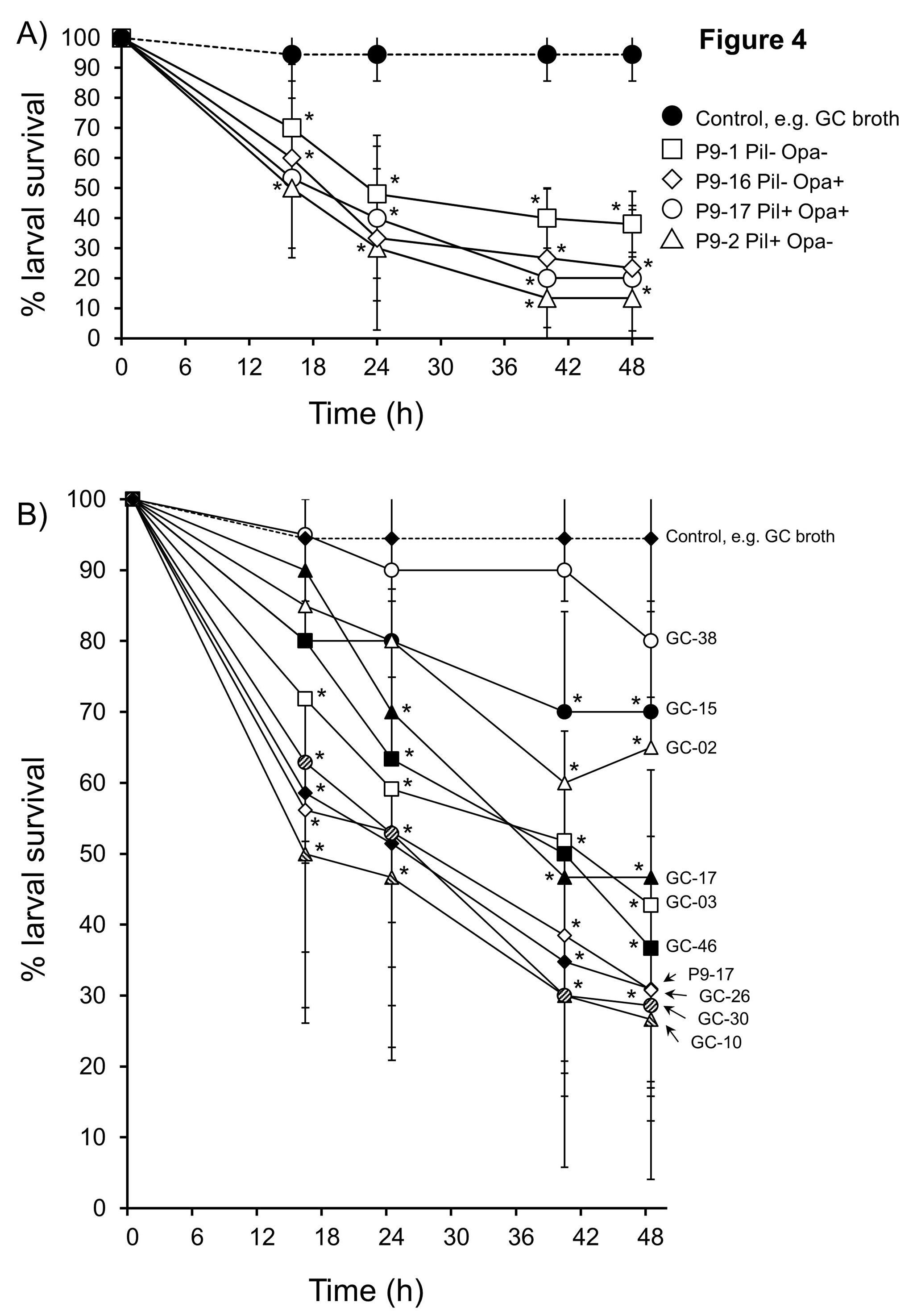

Supplement: Supplemental Material [file KVIR_A_1950269_SM6600.zip › supplementary/Supplementary_Figure_4_new.jpg]

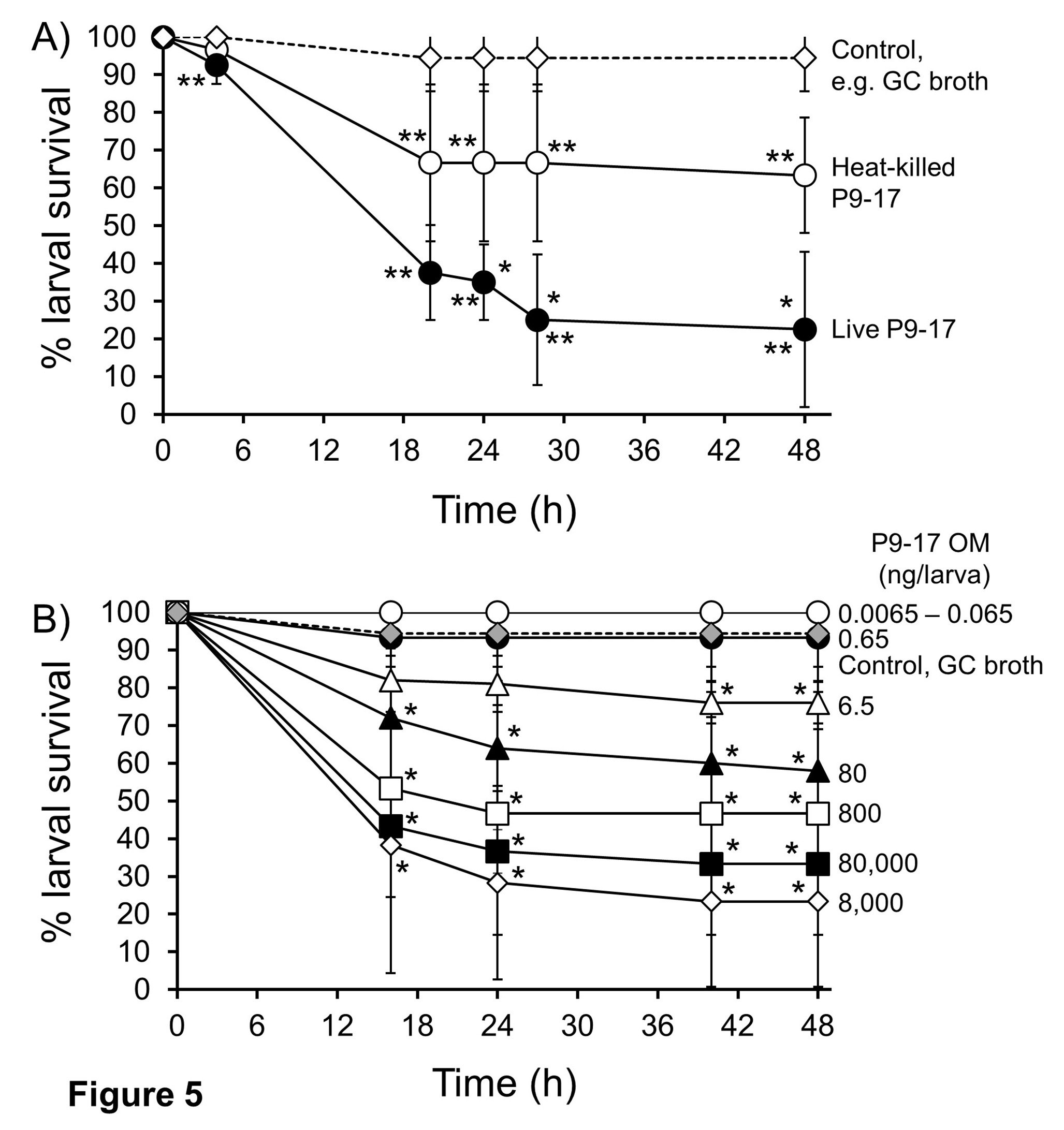

Supplement: Supplemental Material [file KVIR_A_1950269_SM6600.zip › supplementary/Supplementary_Figure_5_new.jpg]

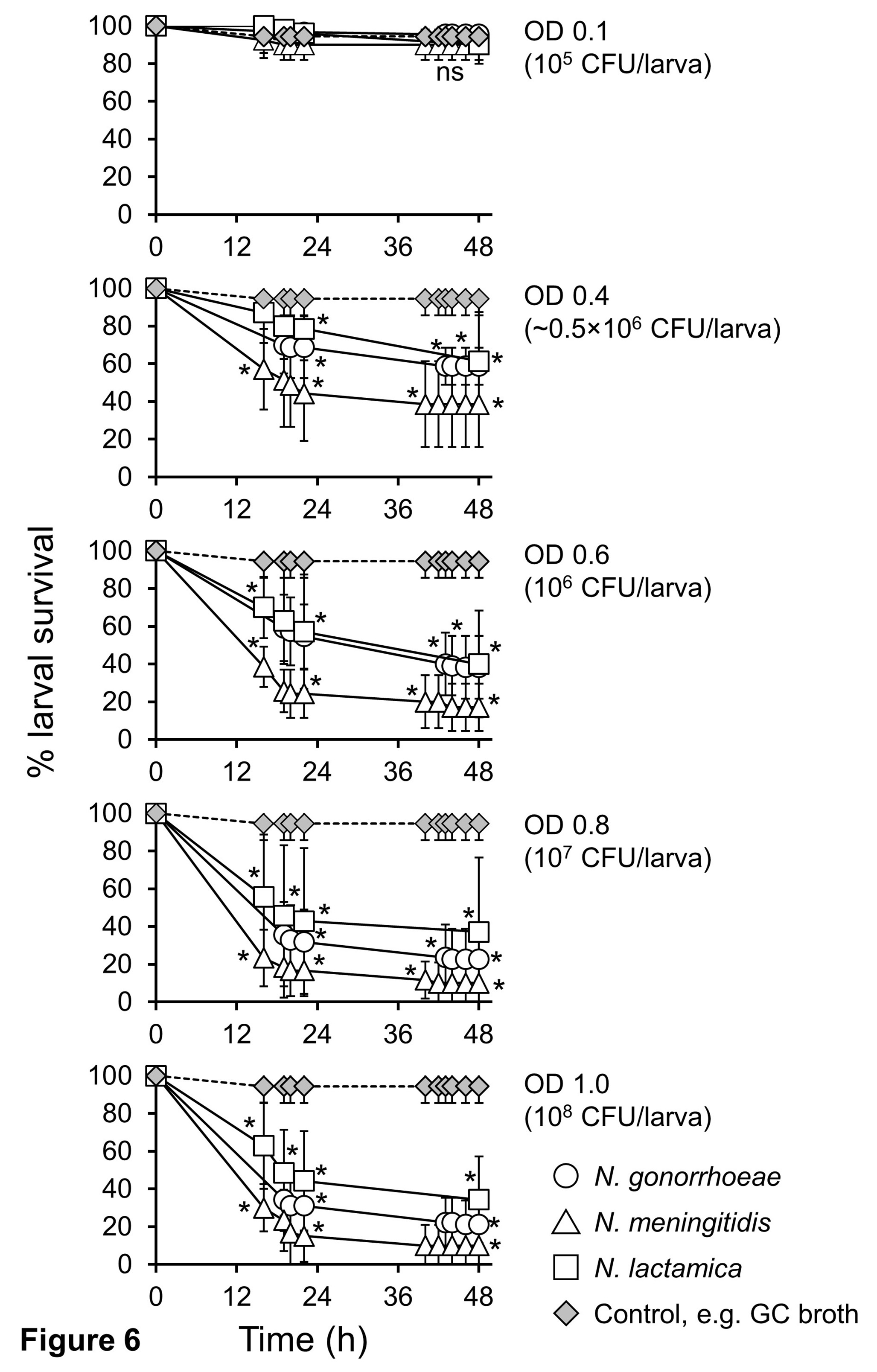

Supplement: Supplemental Material [file KVIR_A_1950269_SM6600.zip › supplementary/Supplementary_Figure_6_new.jpg]

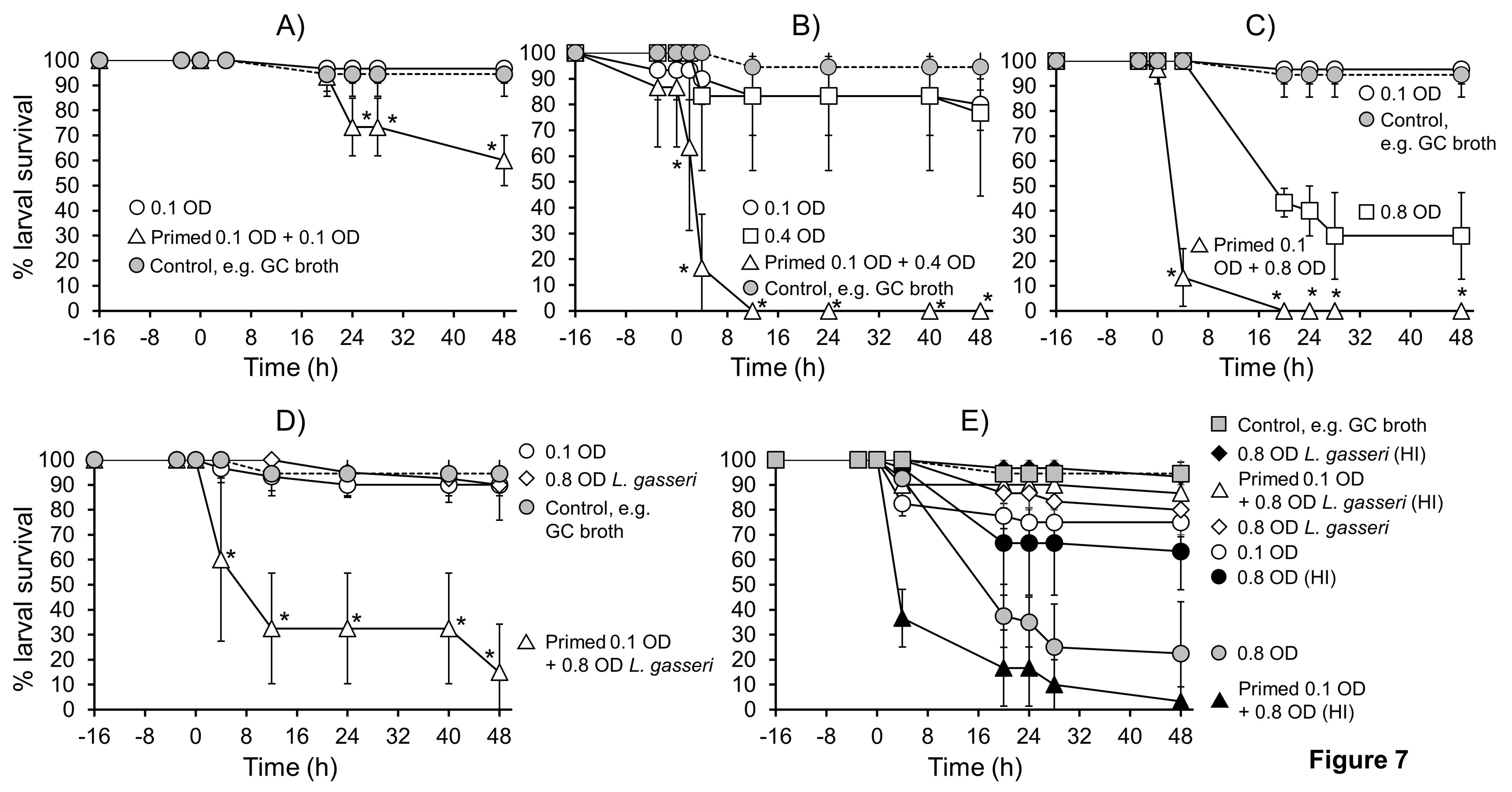

Supplement: Supplemental Material [file KVIR_A_1950269_SM6600.zip › supplementary/Supplementary_Figure_7_new.jpg]

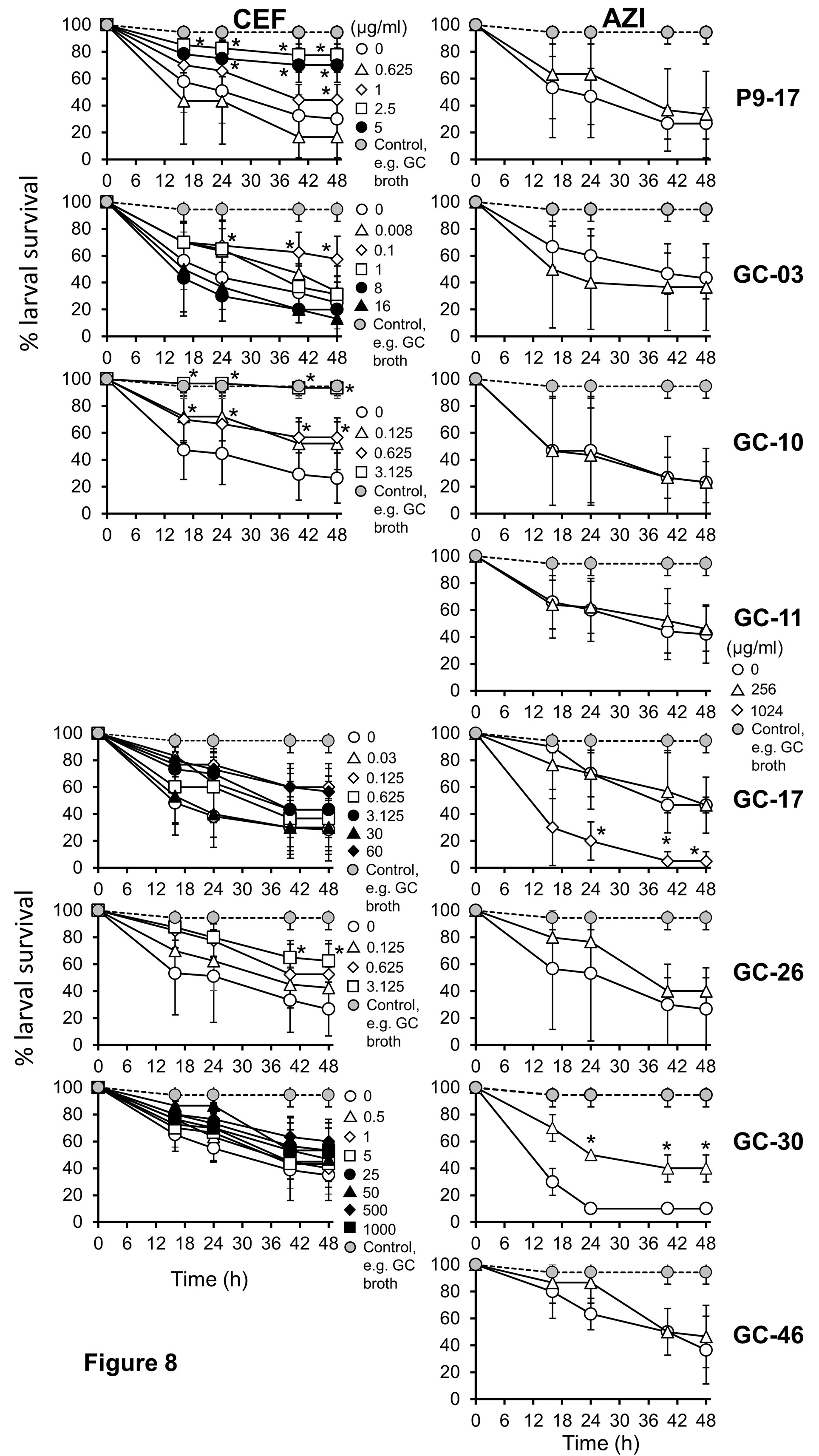

Supplement: Supplemental Material [file KVIR_A_1950269_SM6600.zip › supplementary/Supplementary_Figure_8_new.jpg]
